# Gene Regulatory Networks in Peripheral Mononuclear Cells Reveals Critical Regulatory Modules and Regulators of Multiple Sclerosis

**DOI:** 10.1038/s41598-019-49124-x

**Published:** 2019-09-04

**Authors:** Perumal Gnanakkumaar, Ram Murugesan, Shiek S. S. J. Ahmed

**Affiliations:** 1 Faculty of Allied Health Sciences, Chettinad Academy of Research and Education (CARE), Kelambakkam, 603103 India; 2 Drug Discovery Lab, Faculty of Allied Health Sciences, Chettinad Hospital & Research Institute (CHRI), Chettinad Academy of Research and Education (CARE), Kelambakkam, 603103 India

**Keywords:** Myelin biology and repair, Regulatory networks

## Abstract

Multiple sclerosis (MS) is a complex, demyelinating disease with the involvement of autoimmunity and neurodegeneration. Increasing efforts have been made towards identifying the diagnostic markers to differentiate the classes of MS from other similar neurological conditions. Using a systems biology approach, we constructed four types of gene regulatory networks (GRNs) involved in peripheral blood mononuclear cells (PBMCs). The regulatory strength of each GRN across primary progressive MS (PPMS), relapsing-remitting MS (RRMS), secondary progressive MS (SPMS), and control were evaluated by an integrity algorithm. Among the constructed GRNs (referred as TF_gene_miRNA), POU3F2_CDK6_hsa-miR-590-3p, MEIS1_CASC3_hsa-miR-1261, STAT3_OGG1_hsa-miR-298, and TCF4_FMR1_hsa-miR-301b were top-ranked and differentially regulated in all classes of MS compared to control. These GRNs showed potential involvement in regulating various molecular pathways such as interleukin, integrin, glypican, sphingosine phosphate, androgen, and Wnt signaling pathways. For validation, the qPCR analysis of the GRN components (TFs, gene, and miRNAs) in PBMCs of healthy controls (n = 30), RRMS (n = 14), PPMS (n = 13) and SPMS (n = 12) were carried out. Real-time expression analysis of GRNs showed a similar regulatory pattern as derived from our systems biology approach. Also, our study provided several novel GRNs that regulate unique and common molecular mechanisms between MS conditions. Hence, these regulatory components of GRNs will help to understand the disease mechanism across MS classes and further insight may though light towards diagnosis.

## Introduction

Multiple sclerosis (MS) is a complex demyelinating disease that affects the central nervous system (CNS). Based on the progression, MS is classified as relapsing-remitting (RRMS), primary progressive (PPMS) and secondary progressive (SPMS). Nearly 2.5 million people are affected globally, of which the majority exhibit relapsing-remitting MS. The etiology and molecular mechanisms of MS are largely unknown^1^. Investigations suggest that both genetic and epigenetic factors play a crucial role in disease susceptibility^2,3^. Gene expression changes are reported in the blood and brain of multiple sclerosis patients that are associated with autoimmune and neurodegenerative process^4^. International Multiple Sclerosis Genetics Consortium has performed a genome-wide association study to explore genetic predispositions of MS, which suggests multiple disease-associated loci^5^. Dysregulation of the regulatory mechanism at MS loci has been noticed to alter the expression of genes related to MS^6^.

Gene regulatory network (GRN) plays a vital role in normal cellular processes such as metabolism, cell differentiation, cell cycle, and cell signaling. GRN regulates gene expression through ‘*cis’* and ‘*trans’* regulatory elements such as microRNA (miRNA) and transcription factor (TF). miRNA is a single-stranded, non-coding RNA with ~22 nucleotide bases binds at the 3′ untranslated region (UTR) of the targeted gene (mRNA) for degradation^7^. Over 60% of mammalian genes are controlled by miRNAs^8^. Each miRNA regulates hundreds of its target genes. Change in miRNA expression plays an important role in the development and progression of MS. Particularly, altered expression of miR-326, miR-155, miR-146a, miR-146b, miR-142-3p, and miR-21 dysregulate interleukins and apoptotic process in MS^9^. Also, miRNA and TF co-regulate each other to form a regulatory network that controls cellular gene expression. TF regulates gene at the transcriptional level within the nucleus, whereas the miRNA is post-transcriptionally active at the cytoplasm. Many of such, dysregulating GRNs are reported in Alzheimer’s disease, Parkinson’s disease and Schizophrenia^10–12^. Understanding the regulatory network will enhance the knowledge of cellular mechanism, which may light towards the diagnosis and treatment for the disease. Several efforts have been made to understand the GRNs in multiple sclerosis^13–16^. However, their studies are compromised while describing the type of GRNs and their regulatory strength contributing to RRMS, PPMS, and SPMS.

**Figure 1:**
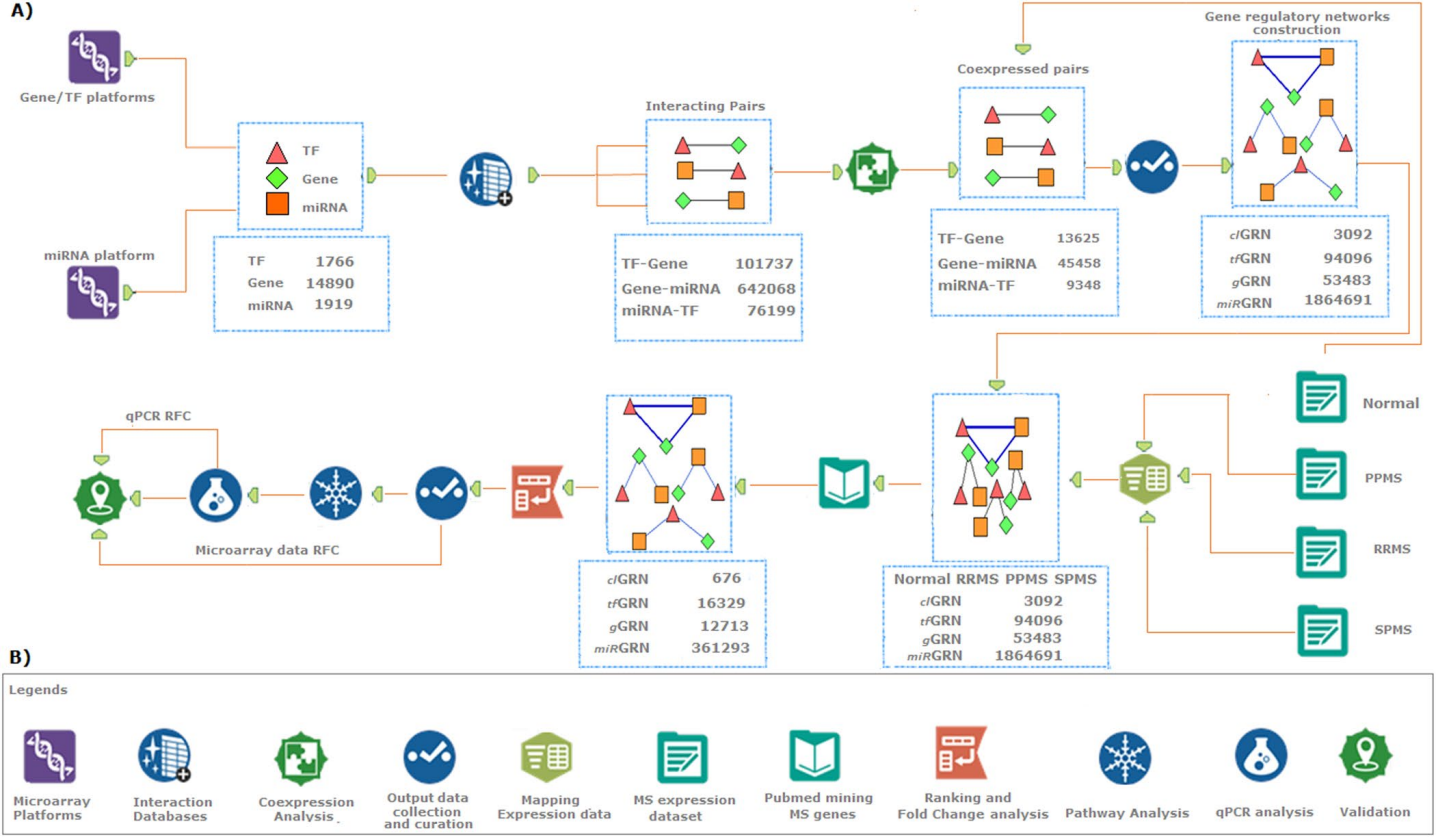
Schematic representation of the overall strategy used in this study. (**A**) Workflow explaining the collection, construction, and validation of gene regulatory networks. (**B**) Components used in the workflow.

In this study (Fig. 1) we develop, four types of GRNs across three MS conditions to demonstrate its molecular pathogenesis. We integrate the gene and miRNA expression profile of PBMCs with a systems biology approach to create GRN based on sequence and co-expressed interaction of TF, gene, and miRNA. An integrity algorithm is implemented to rank significant GRNs based on network integrity. Our approach identifies a diverse range of the regulatory network that explains the common and unique GRNs between the MS conditions. These GRNs regulate several previously known and unknown molecular pathways involved in RRMS, PPMS, and SPMS. Also, qPCR analysis of selected GRN components (TF, gene, and miRNA) confirm the differential regulation in PBMCs of RRMS, PPMS, and SPMS compared to healthy controls. Overall, our study elaborates and highlights the involvement of GRNs in PBMCs of multiple sclerosis which is essential to understand the disease pathogenesis that may throw light towards biomarkers for diagnosis.

## Results

### Co-expressed interaction and gene regulatory network

**Figure 2:**
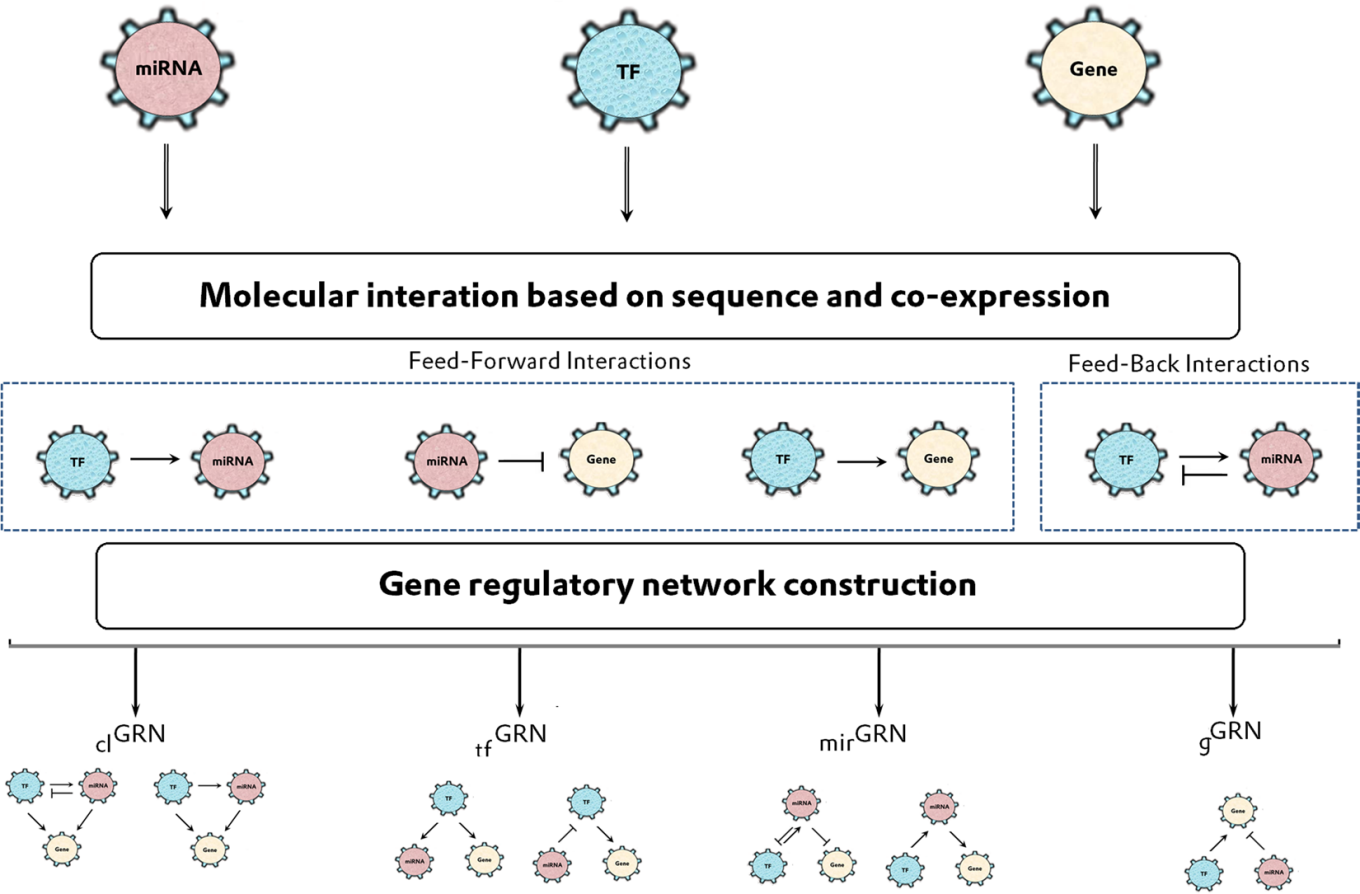
Workflow for the construction of gene regulatory motifs (1) _cl_GRN: closed-loop convergent network; (2) _g_GRN: a common gene GRN; (3) _tf_GRN: a common TF GRN and (4) _miR_GRN: a common miRNA GRN.

To construct the PBMC based gene regulatory network, the gene, TF, and miRNA expressed in human PBMCs were collected from microarray platforms. All extracted data were curated and converted into an official symbol using the Hugo gene nomenclature committee (HGNC) database to have 14980 genes, 1766 TFs, and 1919 miRNAs. From the curated list, the interactions between TFs, genes, and miRNAs were retrieved. A total of 820004 feed-forward and feed-back co-expressed interactions were identified. To validate these interactions, the Pearson correlation analysis was executed using microarray expression data of healthy controls. The microarray datasets E-MTAB-358 (gene) and E-MTAB-359 (miRNA) were obtained from the Array Express repository. Of the 820004 analyzed interactions, 155611 were significantly co-expressed to have 2016172 GRNs based on their common molecular entities, as described in the methodology (Fig. 2). Among 2016172 GRNs, 3092, 53483, 94906 and 1864691 were attributed to _*cl*_GRNs, _*g*_GRNs, _*tf*_GRNs, and _*miR*_GRNs, respectively. These GRNs were mapped with the microarray expression data of healthy controls, RRMS, SPMS, and PPMS (4*2016172). Further, the GRNs were selected, which contains MS-associated genes and TFs that were obtained from the text-mining approach.

### Text-mining

In text-mining, we retrieved 56335 abstracts from the PubMed database using a combination of keywords related to multiple sclerosis (articles published from January 2000 to May 2017). Using an in-house R-script, 2022 genes and 473 TFs associated with MS were extracted from the PubMed abstracts. Among 2016172, the GRNs containing MS-associated text-mined genes and TFs were filtered to have 391011 (_*cl*_GRNs = 676, _*g*_GRNs = 12713, _*tf*_GRNs = 16329 and _*miR*_GRNs = 361293) GRNs in each MS condition and control.

### GRN integrity scoring algorithm

**Figure 3:**
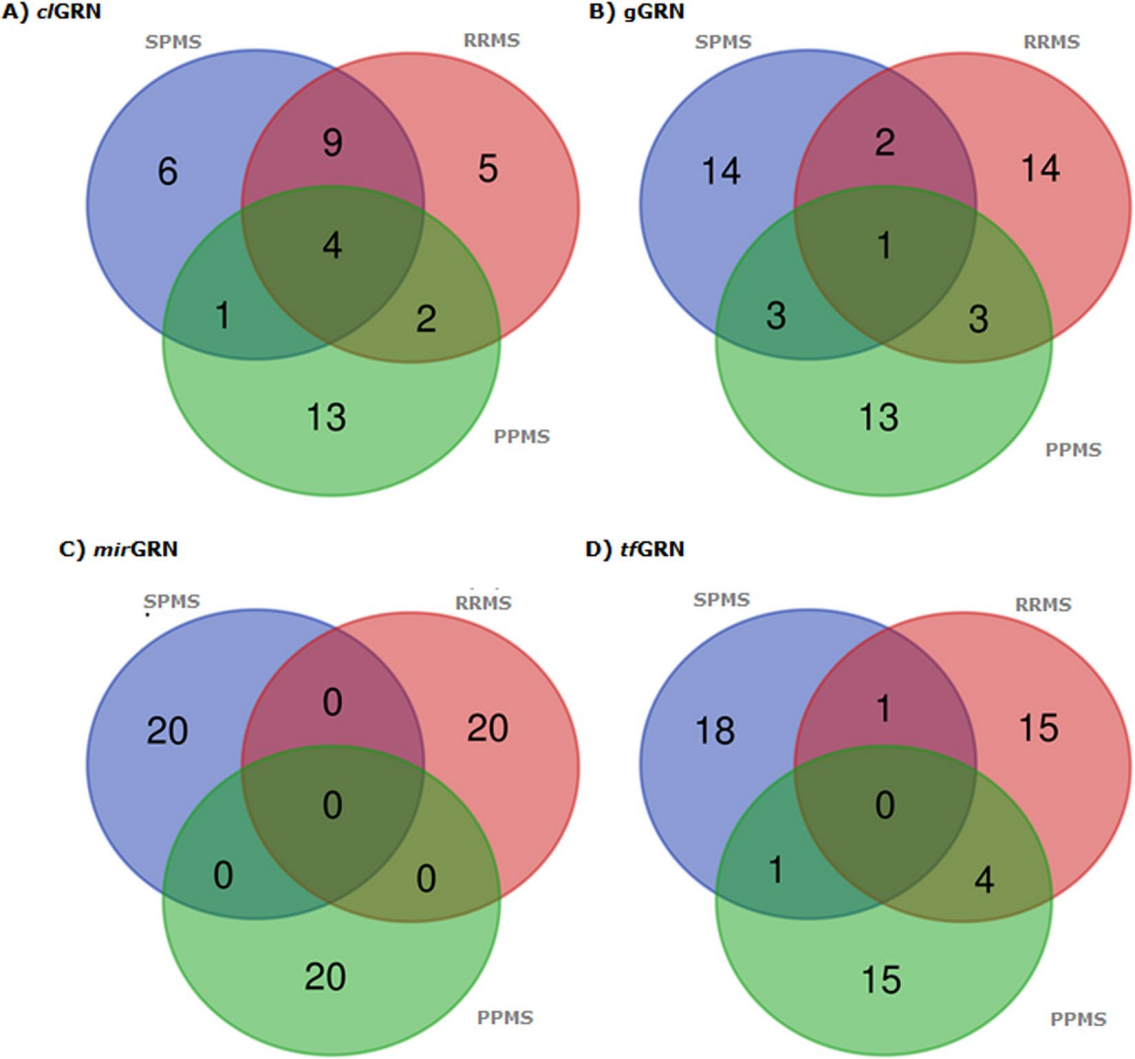
Venn diagrams representing the common and unique GRNs across disease conditions. (**A**) _cl_GRNs, (**B**) _g_GRNs, (**C**) _miR_GRNs and (**D**) _tf_GRNs. The green circle represents PPMS, the pink circle represents RRMS and the blue circle represents SPMS.

The integrity algorithmic score (N) was calculated to determine the regulatory strength of 391011 GRNs in control, RRMS, PPMS, and SPMS (4*391011 = 1564044). The regulatory strength of GRN was ranged from 5.92 to 29.93 for control. In RRMS, it was ranged from 6.38 to 22.39. Similarly, for SPMS and PPMS regulatory strength was ranged between 6.24 to 24.10 and 6.19 to 24.5, respectively. Further, the regulatory fold change (RFC) was evaluated for each GRN between, a) control vs RRMS, b) control vs PPMS and c) control vs SPMS. Based on the RFC, a twenty top-ranked (ten up and ten down) differentially regulated GRNs in RRMS, PPMS, and SPMS were identified. A total of 240 differentially regulating top-rank GRNs were identified, containing 20 for each GRN type (_*cl*_GRNs, _*g*_GRNs, _*tf*_GRNs, and _*miR*_GRNs) across three MS classes (80*3 = 240 GRNs) (Supplementary information 2). Among 240 GRNs, several were unique and common between MS conditions (Fig. 3). For instance, of 60 differentially regulating top-ranked _*cl*_GRNs, POU3F2_CDK6_hsa-miR-590-3p, MEIS1_CASC3_hsa-miR-1261, STAT3_OGG1_hsa-miR-298 and TCF4_FMR1_hsa-miR-301b were commonly noticed in all MS conditions. The POU3F2_CDK6_hsa-miR-590-3p was down-regulated in PPMS and up-regulated in RRMS and SPMS. Whereas, the other three GRNs were down-regulated in all the conditions.

### Functional enrichment analysis

**Figure 4:**
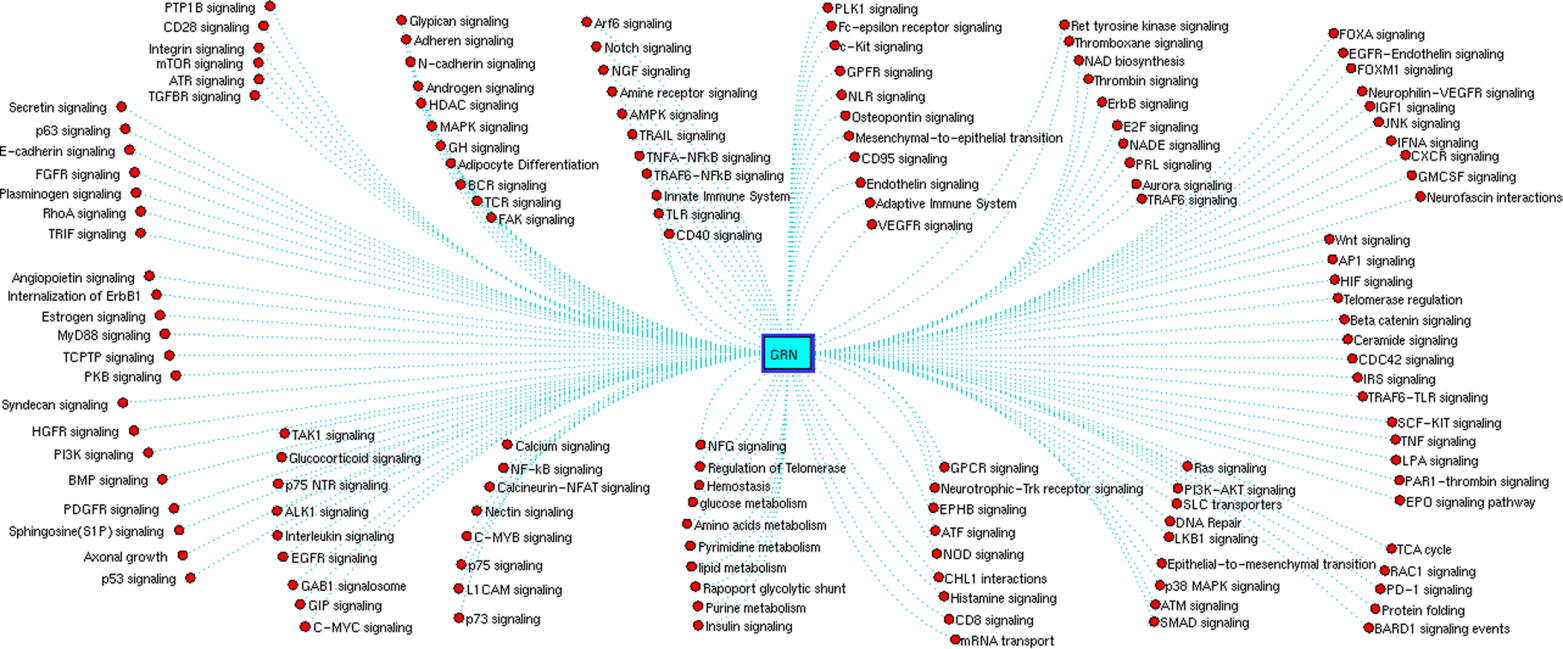
GRNs regulating 144 pathways associated with multiple sclerosis.

Functional analysis of 240 GRNs showed involvement in regulating 144 pathways (Fig. 4). To our knowledge of these 144, a few were previously reported while others were noticed novel to MS conditions. Although few molecular pathways were well reported in MS, our study provides the functional insight about the regulators that contribute to these pathways (Supplementary information 2). For instance, 51 pathways were regulated by five GRNs (four _*cl*_GRNs and one _*g*_GRN) which were commonly noticed between PPMS, SPMS, and RRMS. Similarly, 67 pathways were regulated by eight common GRNs of SPMS and RRMS. Whereas, nine common GRNs of RRMS and PPMS regulate 63 molecular pathways. Also, three GRNs of PPMS and SPMS regulate 33 molecular pathways. Most of these GRNs regulate T-cells immune responses, oligodendrocyte maturation, androgen signaling, axon myelination, and hormonal signaling (Fig. 4). In particular, POU3F2_CDK6_hsa-miR-590-3p, MEIS1_CASC3_hsa-miR-1261, STAT3_OGG1_hsa-miR-298, and TCF4_FMR1_hsa-miR-301b regulate interleukin signaling, integrin signaling, glypican signaling, sphingosine phosphate signaling, androgen signaling, and Wnt signaling mechanism (Supplementary information 2). Understanding the potential involvement of the four _*cl*_GRNs in all MS conditions, their components (TF, gene, and miRNA) may hold good as candidate markers. Hence, the relative expression levels of STAT3, POU3F2, MEIS1, TCF4, CDK6, CASC3, OGG1, FMR1, hsa-miR-590-3p, hsa-miR-1261, hsa-miR-298 and hsa-miR-310 were quantified using qPCR (2 − ∆Ct) in PBMCs of healthy controls and MS patients.

### Real-time validation of the top-ranked GRNs

**Figure 5:**
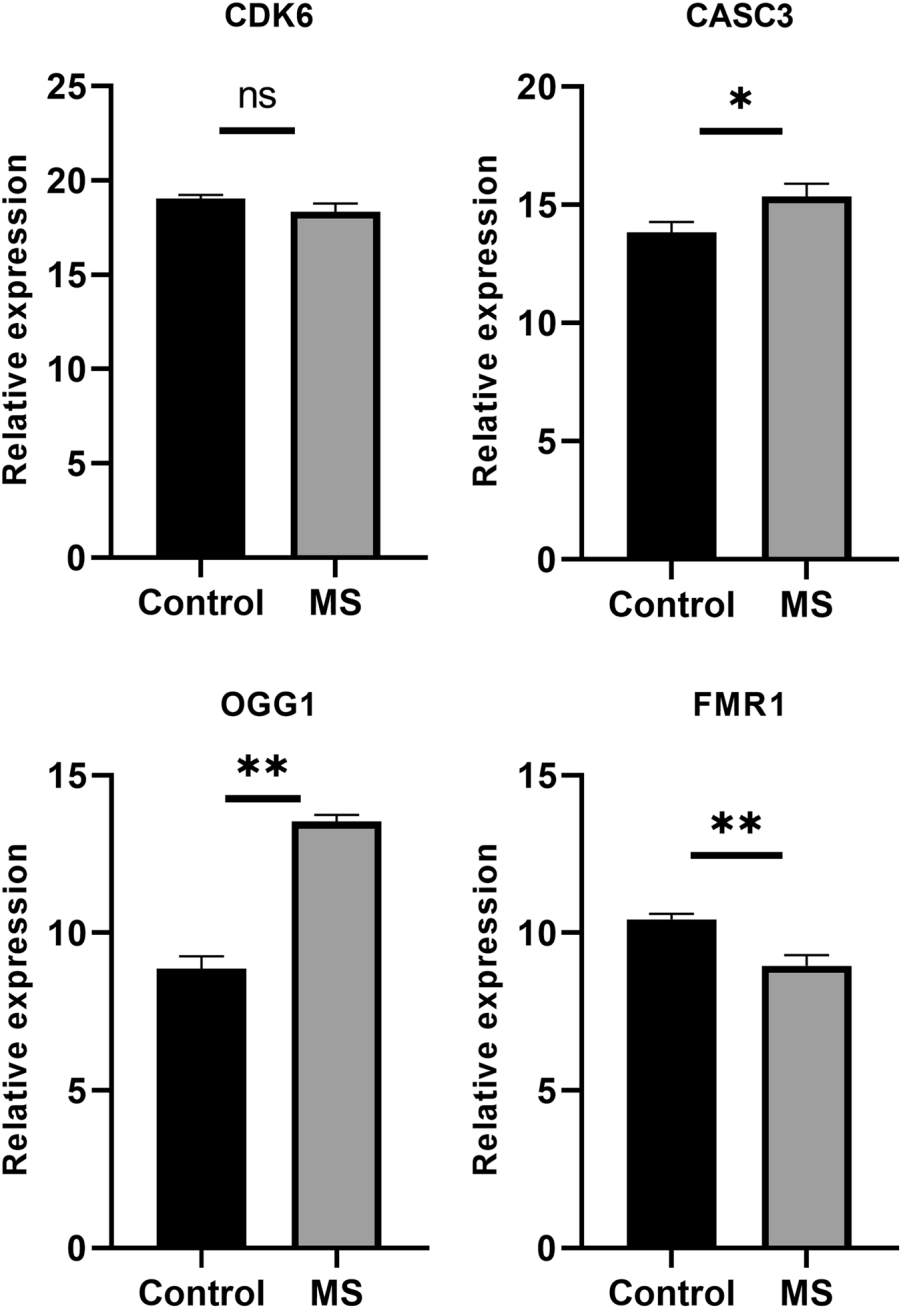
Relative expression of the genes in control and MS were plotted. GAPDH gene was used as an internal control to calculate relative expression. Vertical bars represent the mean ± standard error of the mean. Asterisks indicate statistical significant (*p ≤ 0.05, **p ≤ 0.01 and ns: no significant).

**Figure 6:**
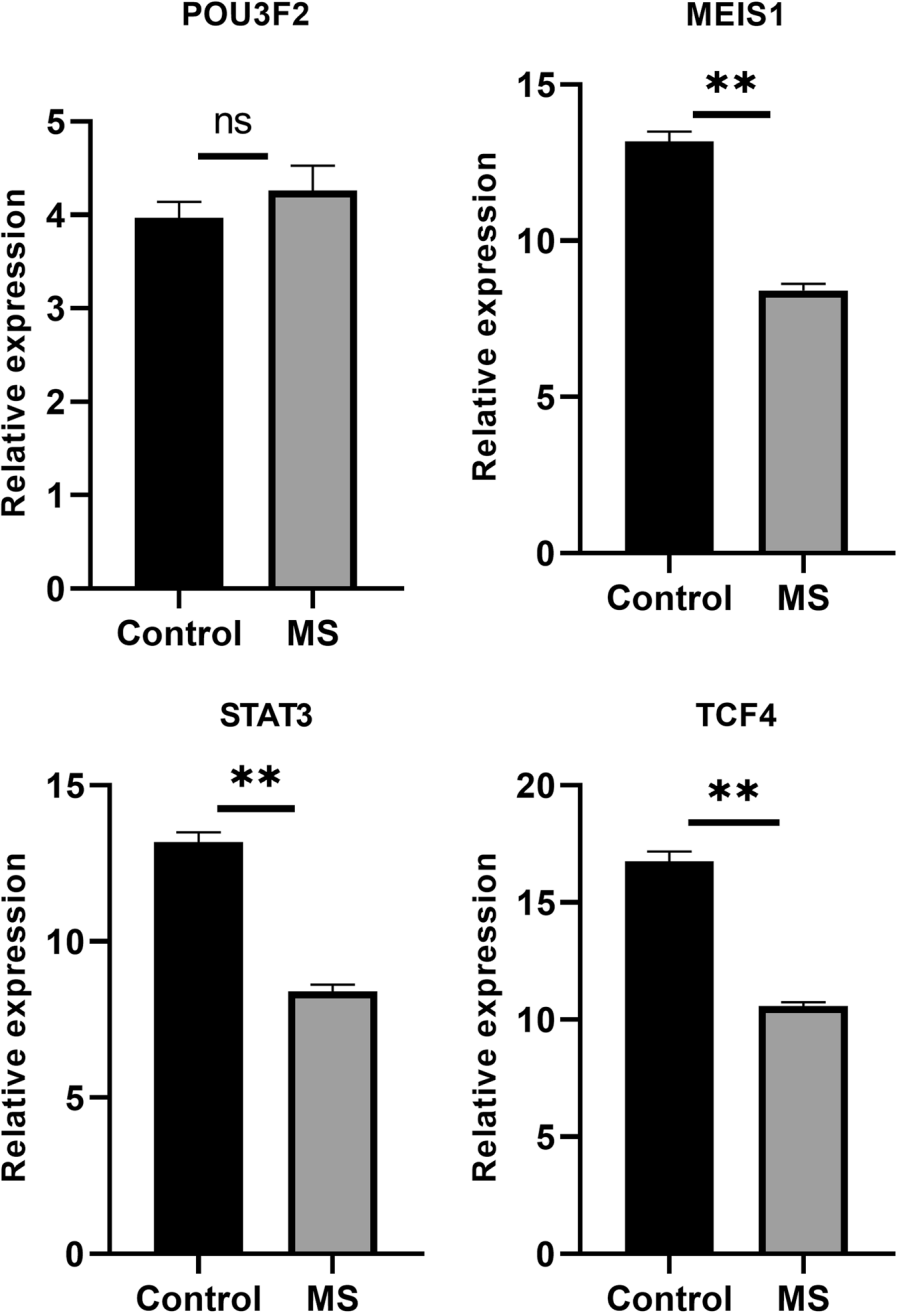
Relative expressions of the selected transcription factors between control and MS were plotted. GAPDH gene was used as an internal control to calculate relative expression. Data are expressed as the mean ± standard error of the mean. Asterisks represent statistical significant (*p ≤ 0.05, **p ≤ 0.01 and ns: no significant).

**Figure 7:**
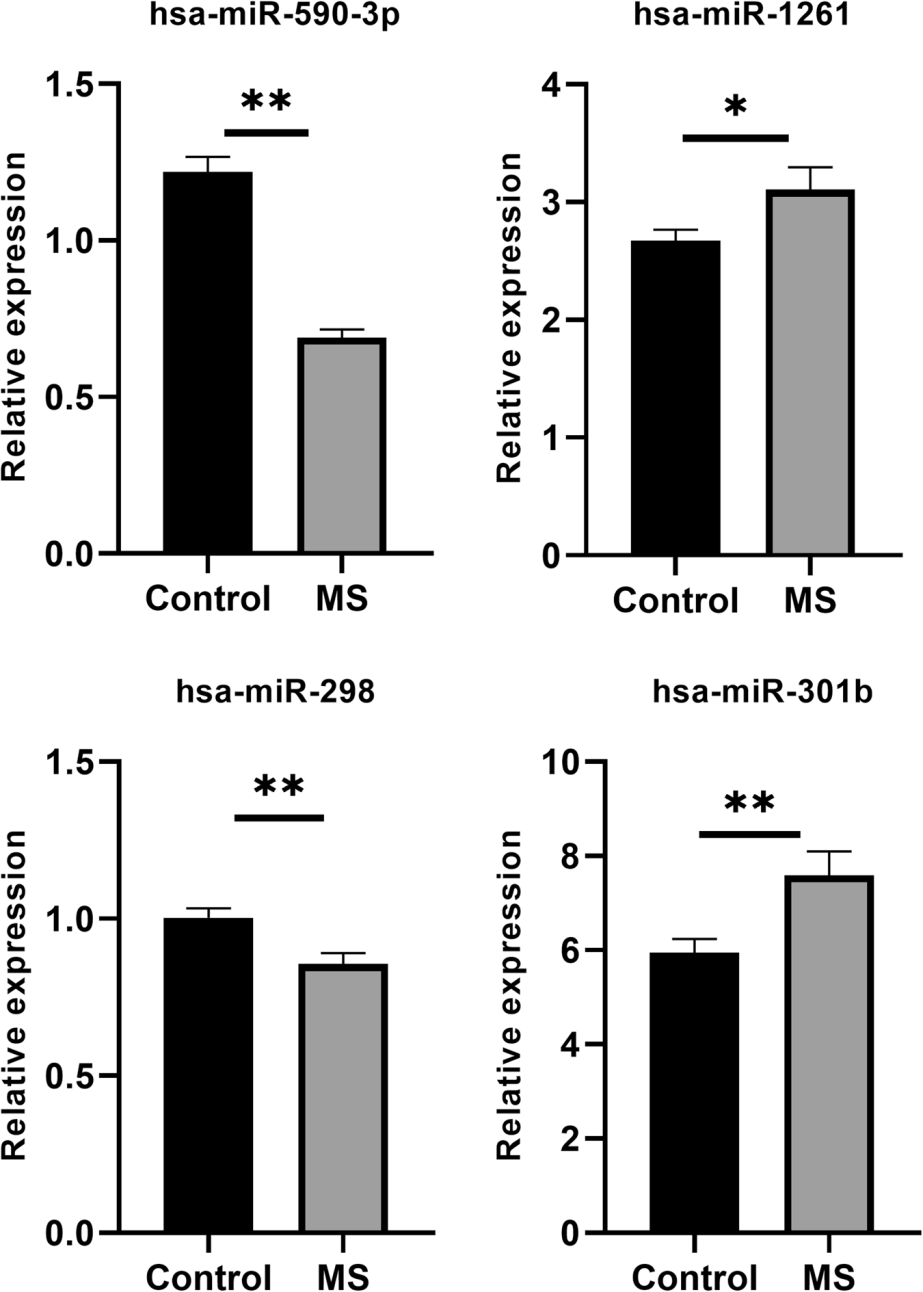
Relative expressions of the selected miRNAs between control and MS were plotted. U6 snRNA miRNA is used as an internal control to calculate a relative expression. Data are expressed as the mean ± standard error of the mean. Asterisks indicate statistical significant (*p ≤ 0.05, **p ≤ 0.01 and ns: no significant).

We observed a significant (p-value ≤ 0.05) up-regulation of OGG1, CASC3, hsa-miR-1261 and hsa-miR-301b and down-regulation of FMR1, MEIS1, STAT3, TCF4, hsa-miR-298, and hsa-miR-590-3p in pooled MS (RRMS + SPMS + PPMS) compared with healthy controls (Figs 5–7). Further, the sub-group analysis (Supplementary information 1) based on MS conditions showed similar trends as pooled MS, except for CASC3 and hsa-miR-1261. Significant (p-value ≤ 0.05) down-regulation of hsa-miR-1261 was noticed in RRMS and up-regulation was observed in PPMS and SPMS. Whereas, CASC3 was down-regulated in SPMS and up-regulated in PPMS and RRMS with p-value ≤ 0.05. Alternatively, POU3F2 and CDK6 did not show minimal statistical significance (p-value ≤ 0.05) in both pooled and sub-group analysis.

### GRN regulation with real-time expression

The integrative score was calculated using the qPCR expression of the TFs, genes, and miRNAs for the top-ranked four _*cl*_GRNs in control, RRMS, PPMS, and SPMS. Based on the score, RFCs were calculated which showed down-regulation of POU3F2_CDK6_hsa-miR-590-3p in PPMS and up-regulation in RRMS and SPMS. Whereas, other GRNs were down-regulated in all MS conditions. The accuracy of the integrity algorithm was determined by comparing the regulatory pattern between the calculated RFC of experimental qPCR data and microarray data (E-MTAB-358 and E-MTAB-359). All four GRNs showed a similar regulatory pattern in RRMS, PPMS, and SPMS between qPCR and microarray data. Although, POU3F2_CDK6_hsa-miR-590-3p exhibit similar regulatory pattern, the qPCR expression of POU3F2 and CDK6 were statistically insignificant (p-value ≤ 0.05) in all MS conditions. Overall, the analysis of four GRNs across three MS conditions (4*3 = 12) showed nine truly classified output except for POU3F2_CDK6_hsa-miR-590-3p in RRMS, PPMS, and SPMS, which show 75% accuracy of the integrity algorithm.

## Discussion

Recent development in the technologies allow us to dissect and describe the molecular function of the cell from a single functional molecule to complex biological pathways. Several genetic analyses show the importance of miRNA and transcription factor in regulating gene expression in normal physiological conditions. For instance, both miRNA and transcription factors are involved in the regulatory process of brain development, neuronal differentiation, and synaptic plasticity. Thus, understanding the role of regulatory network in the pathological state will aid in the development of both diagnostic markers and new therapeutic strategies. Although several studies generate and discuss the GRNs in multiple sclerosis, there are few limitations that had a potential impact on the biological relevance^13–16^. Particularly, in most of the studies (1) GRNs were constructed using a heterogeneous microarray dataset from the various populations. (2) A few types of GRNs were focused, (3) No significant exploration was made to differentiate GRNs based on MS conditions (RRMS, SPMS, and PPMS). (4) The regulatory strength of the GRN was not explicitly defined, which is important in determining the difference in the expression levels of genes in MS. In this juncture, our approach involved in constructing GRNs from the homogenous population avoids experimental bias and population-based expression variation. Using FF and FB interactions, four types of GRNs were constructed for three MS conditions. Also, integrative algorithm and regulatory fold change were implemented to describe the regulatory strength of GRNs in RRMS, SPMS and PPMS compared to healthy controls. In addtion, functional enrichment analysis of GRNs showed several previously un-notified regulatory network of MS-associated molecular pathway mechanisms.

Overall, our analyses identified several promising gene regulatory networks that are unique and common between RRMS, PPMS, and SPMS. Of the identified _*cl*_GRNs, STAT3_OGG1_hsa-miR-298 was noticed top-ranked and common in all three classes of MS. STAT3 transcription factor regulates genes involved in differentiation, proliferation, apoptosis, innate and adaptive immune responses. In particular, STAT3 is activated by the Janus Kinase pathway in response to cytokines that induce inflammatory mechanisms. Also, the knockout mice study of IL-6−/− and STAT3−/− develops encephalomyelitis resistant, which suggests the role of STAT3 in neuropathogenesis^17^. Additionally, a genetic study has shown the risk association of MS in the German population with rs744166 and rs2293152 polymorphism in STAT3^18^. STAT3 was implicated in inflammatory neurodegenerative conditions such as Alzheimer, Parkinson, and Huntington disease^19,20^. In our analysis, STAT3 was noticed to activate OGG1 and hsa-miR-298. The OGG1 encodes 8-oxoguanine DNA glycosylase that involved DNA repair mechanism^21^. A recent study by Roya *et al*. (2017) reported the significant up-regulation of OGG1 in RRMS^22^. Additionally, Karahalil *et al*. (2015) suggest the risk association of OGG1 polymorphism (Ser326Cys) in developing multiple sclerosis^23^. The above studies are in accordance with our findings, confirming the contribution of OGG1 in MS pathogenesis. To our knowledge, there is no literature evidence suggesting the involvement of hsa-miR-298 in MS. However, the report of Dai *et al*. 2007, describes the association of hsa-miR-298 in autoimmune conditions such as systemic lupus erythematosus^24^.

Similar to STAT3 GRN, the TCF4_FMR1_hsa-miR-301b was noticed as one of the top-ranked _*cl*_GRN in all three classes of MS. TCF4 is the potential transcription factor mediates Wnt signaling pathway that associated with infiltration of immune cells in multiple sclerosis. Several studies have reported the significant role of TCF4 in the development of oligodendrocytes and myelination^25–27^. TCF4 regulates the expression of myelin-related genes such as CNPase, MBP, and PLP of neurons. Experimental study of TCF4 null mice showed reduced expression of CNPase, MBP, and PLP in the brain that may cause dysregulation of the myelination process^28–30^. In our analysis, TCF4 was noticed to activate fragile X mental retardation 1 (FMR1) gene, which is associated with the neurodegenerative condition such as Fragile X-associated tremor ataxia syndrome (FXTAS). In myelin-producing oligodendrocytes, FMR1 interacts with MBP that regulates CNS myelination. CNS demyelination is one of the notable factors in neurological diseases, including MS^31^. Several clinical studies have showed that patients with fragile X associated tremor/ataxia syndrome were susceptible to MS^32,33^. In addition, FMR1 and TCF4 were observed to regulate hsa-miR-301b. To our knowledge, no study has shown the influence of hsa-miR-301b in MS. However, the hsa-miR-301b belonging mir-130 family^34^ has implicated in most of the neuroinflammatory conditions^35^.

MEIS1 (transcription factor) is a Meis homeobox 1 protein belongs to TALE homeodomain family, which forms a _*cl*_GRN with CASC3 and hsa-miR-1261. Experimental study of a transgenic mouse with the rs12469063 variant of MEIS1 shows an involvement in the neuronal development process^36^. Although, there is no direct association of MEIS1 with multiple sclerosis, the rs2300478 polymorphism of MEIS1 is proven to contribute to neurological diseases^37^. Similarly, Jang *et al*. (2006) reported the over-expression of CASC3 (also known as MLN51) in autoimmune disease^38^. On the other hand, the role of hsa-miR-1261 in MS has not been widely studied. However, considering the interaction of hsa-miR-1261 with the neurologically associated MEIS1 and CASC3, we suggest MEIS1 regulatory network may have a potential role in the neuropathological process of MS. Similarly, POU3F2 (synonym BRN2) is a member of POU III class of the neuronal transcription factor forms a _*cl*_GRN with CDK6 and miR-590-3p. POU3F2 plays a vital role in the development and differentiation of the neuron. Julien Ghislain *et al*. (2006) showed the involvement of POU3F2 in the process of pro-myelin to myelin transition in Schwann cells^39^. Grafting of Schwann cell in experimental rat showed re-myelination of demyelinated axons in the central nervous system^40^. Down-regulation of POU3F2 suggests dysregulation in myelination processes in MS. In addition, POU3F2 was noticed to regulate CDK6 which activates pro-inflammatory cytokines through NF kappa B and STAT pathways^41,42^. Also, hsa-miR-590 of POU3F2 GRN showed involved in the inflammation process by modulating Th17 cell differentiation in the autoimmune condition of central nervous system^43^.

## Method

### miRNA-TF/gene interactions

To construct human PBMCs based GRNs, the genes, and miRNAs expressed in human PBMCs were retrieved from microarray platforms (GPL95, GPL96, and GPL570) of Gene Expression Omnibus (GEO, http://www.ncbi.nlm.nih.gov/geo/) database. All genes were compiled and converted to the official gene symbols using the HGNC (https://www.genenames.org/) database to avoid duplicates. A curated list of official gene symbols was classified as genes and transcription factors using Tcof and DBD databases^44,45^. Further, the sequence-based interaction between TF and gene was predicted using transcription factor binding sites (TFBS) data of UCSC table browser^46^. To increase the true positive interaction, the Z-score cut-off was set to 2.33. Further, the interaction of TF-gene was confirmed using a Chipbase^47^ database. Similarly, a list of human miRNA was retrieved from the microarray platform (GPL18044). The interaction between miRNA with TF and gene were predicted based on the known promoter sequence following the procedure of Mullany *et al*.^48^. The presence of interactions between miRNA with TF and gene were confirmed from the CircuitDB^49^, TransmiR^50^, puTmiR^51^, miRwalk^52^, miRecords^53^, mirTarbase^54^, Phenomir^55^, and mir2disease^56^ databases. Among these retrieved interactions, we observed three types of regulatory interactions, i) miRNA regulating target gene (miRNA-gene) ii) miRNA regulating TF (miRNA-TF) and, iii) TF regulating miRNA (TF-miRNA). Here the interaction of miRNA-TF and TF-miRNA together act as feedback (FB) interaction, if the same TF and miRNA reciprocally regulate each other (TF ⇋ miRNA). Alternatively, the unidirectional interactions of miRNA-gene, TF-miRNA, and TF-gene were considered as feed-forward (FF) interaction.

### Co-expressed interaction data

The co-expression of TF, gene, and miRNA in FF and FB interactions were validated using Multi-Experiment Matrix (MEM)^57^ and Co-expression Meta-analysis of miRNA Targets (CoMeTa)^58^ databases. MEM generates p-value for each TF-gene interaction; the p-value ≤ 0.05 was considered statistically significant interaction. Similarly, co-expression of TF-miRNA, miRNA-gene, and TF ⇋ miRNA interactions were validated using the CoMeTa database, with a score ≥4. Strict cut-offs for MEM and CoMeTa were followed to minimize false-positive co-expressed interactions.

### GRNs with MS expression data

Besides MEM and CoMeTa, the existence of co-expressed interactions in FBIs and FFIs were cross-validated using microarray expression data of healthy controls. For which, the microarray expression dataset was retrieved from the ArrayExpress database based on the following criteria: (1) Expression profiling should be conducted in PBMC. (2) Gene and miRNA expression profiling should be conducted in the same individual. (3) Enough information should be available for the dataset to classify MS into RRMS, PPMS, and SPMS. (4) The dataset should contain a minimum of three samples in each MS condition, including control. Considering the above criteria, the expression data Accession No. E-MTAB-358 (gene) and E-MTAB-359 (miRNA) were selected from ArrayExpress database^59^ contains 14 controls and 19 MS patients (RRMS 7, SPMS 6, PPMS 6)^60^. The dataset represents miRNA and gene expression profiles from the same individual with the expression data of 35133 genes and 1146 miRNAs. The obtained data were normalized and the co-expression of interactions in FB and FF were confirmed using Pearson’s correlation.

### Gene regulatory network

Based on the co-expressed interactions, four different GRNs (_*cl*_GRN, _*g*_GRN, _*tf*_GRN, and _*miR*_GRN) were constructed with all possible combinations of FB and FF interactions. The closed-loop convergent network (_*cl*_GRN) contains two sub-classes of networks, generated from the interactions (FBIs and FFIs). (1) The interactions with mutual gene target regulated by reciprocal regulators (TF ⇋ miRNA). (2) Interactions with mutual gene target regulated unidirectional regulators (TF-miRNA). Similarly, _*g*_GRN was constructed with the interactions having a common gene to the regulators (TF and miRNA). Likewise, the TF that regulates common gene and miRNA was termed as _*tf*_GRN, whereas the FB and FF interactions with miRNA regulating common TF and genes were denoted as _*miR*_GRN. Further, these four types of gene regulatory networks were used as a template to map expression data of RRMS, SPMS, PPMS, and controls.

### Text mining of MS genes and integrity ranking of GRNs

To extract the MS-associated GRNs, the genes and TFs reported in multiple sclerosis were text-mined using the in-house R-script by collecting abstracts from NCBI PubMed database (https://www.ncbi.nlm.nih.gov/pubmed). The gene regulatory networks of control, RRMS, PPMS and SPMS containing the text mined genes and TFs were selected to determine their regulatory strength. The integrity algorithmic score (N) was calculated for each selected regulatory network of control, RRMS, PPMS, and SPMS.

$$\begin{array}{rcl}N & = & \{[({r}_{tf}\times {e}_{tf})\times ({r}_{mir}\times {e}_{mir})]\\  &  & +[({r}_{mir}\times {e}_{mir})\times ({r}_{gene}\times {e}_{gene})]\\  &  & +[({r}_{gene}\times {e}_{gene})\times ({r}_{tf}\times {e}_{tf})]\}\\ Regulatory\,fold\,Change\,(RFC) & = & \frac{{N}_{MS}}{{N}_{Control}}\end{array}$$

In the integrity algorithmic score (N), the weight of each component in GRN was designated based on their regulatory role in the cellular gene expression process. For instance, TF assigned the highest weight (r_tf_ = 1) for its involvement in initiating the expression of gene and miRNA. Similarly, the gene was designated with moderate weight (r_gene_ = 0.75) by considering its participation with several molecular and cellular processes. Whereas, the regulatory weight of miRNA was assigned as r_mir_ = 0.5 due to its alternate suppression of competing endogenous RNA^61^. In addition to the regulatory weight, the normalized expression values of the gene (e_*gene*_), TF (e_*tf*_) and miRNA (e_*mir*_) were included to determine the regulatory strength of the GRNs in control, RRMS, PPMS, and SPMS. Further, the regulatory fold difference of each GRN between a) *control vs RRMS*, b) *control vs PPMS* and c) *control vs SPMS* were calculated and ranked. The top twenty differentially regulated _*g*_GRNs, _*tf*_GRNs, _*mir*_GRNs, and _*cl*_GRNs were selected (RFC > 1 designated as up-regulation; RFC < 1 determined as down-regulation) in RRMS, PPMS, and SPMS.

### Functional enrichment analysis

The functional enrichment analysis was executed to determine the molecular mechanism of the selected top-ranked GRNs in MS conditions. The TFs, genes, and miRNAs of each GRN were functionally enriched using the FunRich tool^62^. A p-value < 0.05 was considered as the cut-off for enriched pathways. The collected pathways of each molecular entity (TF, gene, and miRNA) was manually curated to have non-redundant pathways. Further, GRN regulating pathway was determined by identifying the commonly representing pathway between TF, gene, and miRNA for each GRN. In addition, expression of top-ranked GRNs (TF, gene, and miRNA) was validated in patients with RRMS, PPMS, SPMS and healthy controls using qPCR.

### Ethics for sample collection

All participants were recruited from the Chettinad Hospital and Research Institute (CHRI), India. The protocol for this study was approved by the Institutional Human Ethics Committee of CHRI (IHEC/04/Sep2014/Desp.no. 420). The written informed consent was obtained from each participant before collecting the samples. All procedures, including sample collection, processing, and analysis, were conducted under the regulations and guidelines of the institutional ethical committee. The neurologist diagnosed the patients with MS based on neurological examination, family and medical history. The McDonald criteria^63^ and expanded disability status scale (EDSS)^64^ were followed to have true-positive MS patients. For the comparative analysis, participants with no sign of neurologic or neuropsychiatric symptoms were taken as a control.

### Inclusion and exclusion criteria for sample collection

Participants (control = 30; RRMS = 14; PPMS = 13 and SPMS = 12) were selected based on the following criteria. Inclusion criteria: (1) the ability to comply with study procedures. (2) Diagnosis based on McDonald criteria and EDSS score. The exclusion criteria include (1) history of HIV infection, immunodeficiency disease and autoimmune diseases other than MS. (2) coexistence of other neurological symptoms. Demographic parameters such as age, gender, disease status, and treatment were recorded before blood collection (Table 1).

**Table 1:** Demographic characteristics of Samples collected.

	Control	Multiple Sclerosis
Number of participants	30	39
Age (years)^¥^	51.8 ± 10.9	46.9 ± 8.7
Gender ratio (male: female)	15:15	16:14
Disease Course (PPMS/RRMS/SPMS)	—	13/14/12
**Treatment**		
Dimethyl fumarate	—	12
Fingolimod	—	14
Teriflunomide	—	13

^¥^Mean; ± Standard error of mean (SEM).

### Sample collection and processing

Peripheral blood (5 ml) was collected from 30 healthy controls (average age 51.8 ± 10.9 years) and 39 multiple sclerosis patients (average age 46.9 ± 8.7 years). Further, PBMCs were isolated using graduated centrifugation over Lymphoprep™ (STEMCELL Technologies, UK). Total RNA was extracted by Trizol method and the quality was assessed using a NanoDrop ND-1000 spectrophotometer. The extracted RNA was further purified into two separate fractions containing small (18–200 bases) and large (>200 bases) using NucleoSpin miRNA, Machery-Nagel kit.

### Gene and miRNA expression

From the large fraction, cDNA was synthesized using SuperScript® III Reverse Transcriptase kit (Life Technologies, NY). SYBR green PCR master mix (Applied Biosystems, CA) was used to perform qRT-PCR on 7900 HT Fast Real-Time PCR system. The miScript II RT Kit, Qiagen and Fast SYBR Green Master Mix, Invitrogen was used to detect the levels of selected miRNA expression following the manufacturer’s protocol. U6 snRNA and GAPDH were used as the internal control for miRNA and mRNA analysis, respectively. The relative expression of the selected gene and miRNA was determined by following the 2 − ∆Ct calculation^65^. The primers of selected TFs, genes, and miRNAs were shown in Table 2. Further, fold change was calculated, and the student’s t-test was performed to analyze the statistical significance between the control and pooled MS (RRMS + PPMS + SPMS). Sub-group analysis, (a) *control vs RRMS*, (b) *control vs PPMS*, and (c) *control vs SPMS* were carried out by comparing appropriate age and gender-matched control for RRMS, PPMS, and SPMS, respectively. The significance of TF, gene, and miRNA in RRMS, SPMS and PPMS were inspected by following similar statistical procedures. Further, the qPCR expression of the genes, TFs, and miRNAs were implemented in the integrity algorithm. We compared the resulting GRNs pattern based on RFCs with microarray data to determine the accuracy of the algorithm.

**Table 2:** Genes and microRNA primer sequence.

GENES/miRNAs	orientation	Primer Sequence
STAT3	*Forward*	ACCCAACAGCCGCCGTAG
	Reverse	CAGACTGGTTGTTTCCATTCAGAT
POU3F2	*Forward*	CCGCAGCGTCTAACCACTAC
	Reverse	GTGGGACAGCGCGGTGATCC
MEIS1	*Forward*	TGACCGTCCATTACGAAACCT
	Reverse	CCAGTCCAACCGAGCAGTAAG
TCF4	*Forward*	ACATGCATGGAATCATTGGA
	Reverse	TGAATGTCTGTTGGCTGAAA
CDK6	*Forward*	CTGAATGCTCTTGCTCCTTT
	Reverse	AAAGTTTTGGTGGTCCTTGA
CASC3	*Forward*	CAAGGAAGGTCGTGCTGGTT
	Reverse	ACCAGACCGGCCACCAT
OGG1	*Forward*	AATTCCAAGGTGTGCGACTG
	Reverse	CGATGTTGTTGTTGGAGGAAC
FMR1	*Forward*	CCCTTCAAAGAGTCGTCCAC
	Reverse	GTGAGATCCCCAGCTGTCTC
GAPDH	*Forward*	AGCCACATCGCTCAGACAC
(House Keeping gene)	Reverse	GCCCAATACGACCAAATCC
hsa-miR-590-3p	*Forward*	GCAGCGCAGTAATTTTATGTATAAG
	Reverse	GCAGCGCAGTAATTTTATGTATAAG
hsa-miR-1261	*Forward*	AAGGCTTTGGCTTATGGGGATATTGTGGTTGATCTGTTCTATCCAGATGACTGAAACTTTCTCCA
	Reverse	GGTCCAGTTTTTTTTTTTTTTTGCT
hsa-miR-298	*Forward*	GCAGAAGCAGGGAGGT
	Reverse	CCAGTTTTTTTTTTTTTTTGGGAGA
hsa-mir-301b	*Forward*	CTCTGACGAGGTTGCACTACTGTGCTCTGAGAAGCAG
	Reverse	CAGTTTTTTTTTTTTTTTGGTCCCA
U6 snRNA	*Forward*	TGGCCCCTGCGCAAGGATG
	Reverse	GTAGGAACGCGTCCCCGG

## Conclusion

In conclusion, our study explores the regulatory behaviors TF, gene, and miRNA as GRNs in MS. Although the public repository data were used, we implemented several levels of data curation to achieve the pathologically relevant GRNs of MS. Our regulatory scoring algorithm of GRN shows consistency with the real-time expression of TF, gene, and miRNA. Further functional enrichment of the GRNs shows several key regulators for known and unknown molecular pathways across three MS conditions. Interestingly, the potential GRNs that regulating hormone, cellular differentiation, and inflammation have been exposed. Few of other GRNs left out several clues and questions to explore its link between MS. Overall, our results pinpoint the dysregulating regulators of neuronal development and neuroinflammatory processes associated with MS which might help towards the development of biomarkers.

## Supplementary information

Supplementary information 1

Supplementary information 2
