# Supplementary material for: Gene Regulatory Networks in Peripheral Mononuclear Cells Reveals Critical Regulatory Modules and Regulators of Multiple Sclerosis
Source: Sci Rep. 2019 Sep 4;9:12732. doi: 10.1038/s41598-019-49124-x (PMC6726613; doi:10.1038/s41598-019-49124-x)
Supplement: Supplementary file 1 — Supplementary information 1 [file 41598_2019_49124_MOESM1_ESM.docx]

**Gene Regulatory Networks in Peripheral Mononuclear Cells Reveals  Critical  Regulatory Modules and Regulators of Multiple Sclerosis**

Perumal Gnanakkumaar, Ram Murugesan, Shiek SSJ Ahmed

Supplement 1: Subgroup analysis of gene and miRNA expression in multiple sclerosis


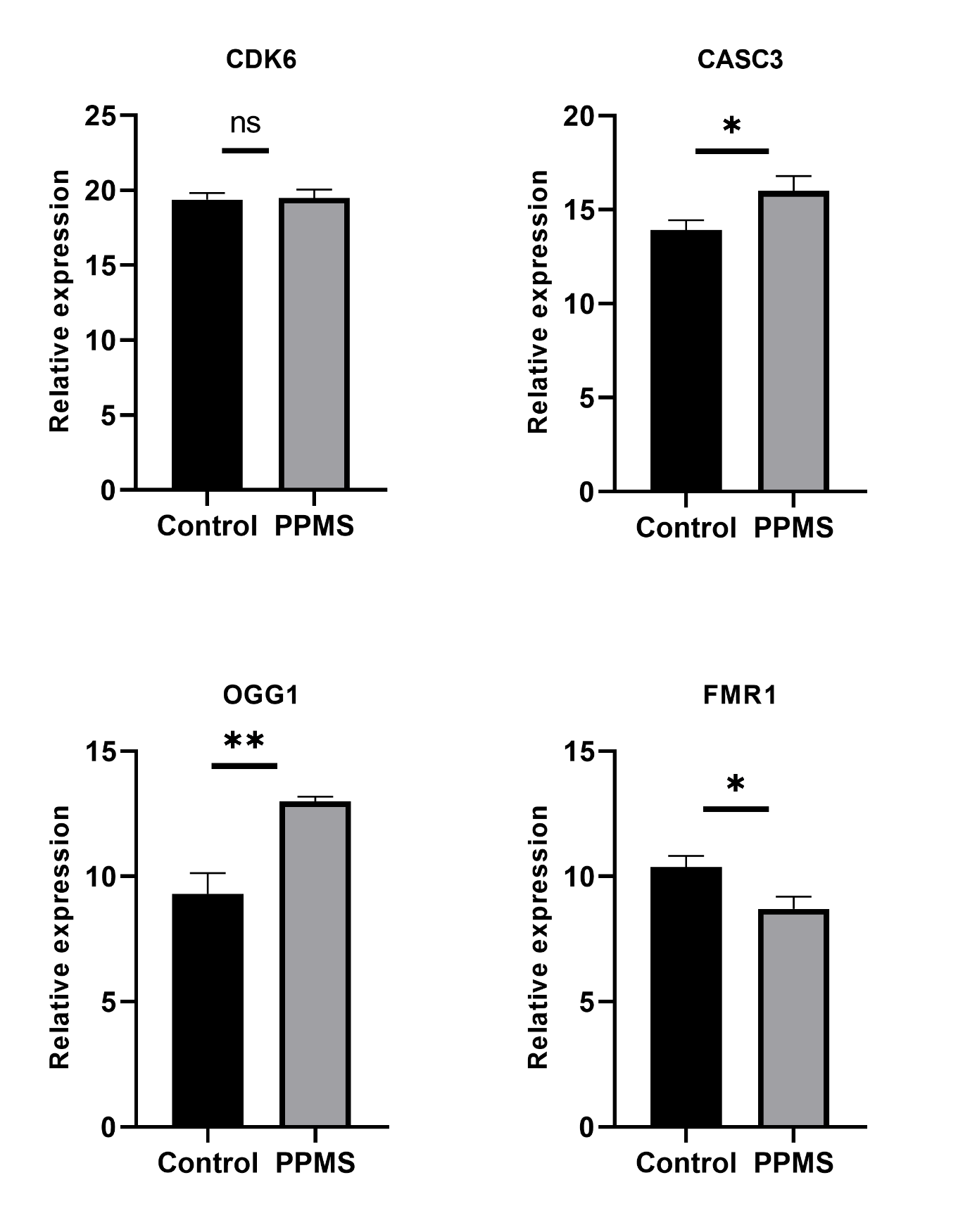


Figure 1.

Subgroup analysis of selected genes in PPMS. Students t test was carried out with age and gender matched controls. Data expressed as means ± standard error of mean. Asterisks indicate statistical difference (*p ≤ 0.05, ** p ≤ 0.01 and ns: no significant).


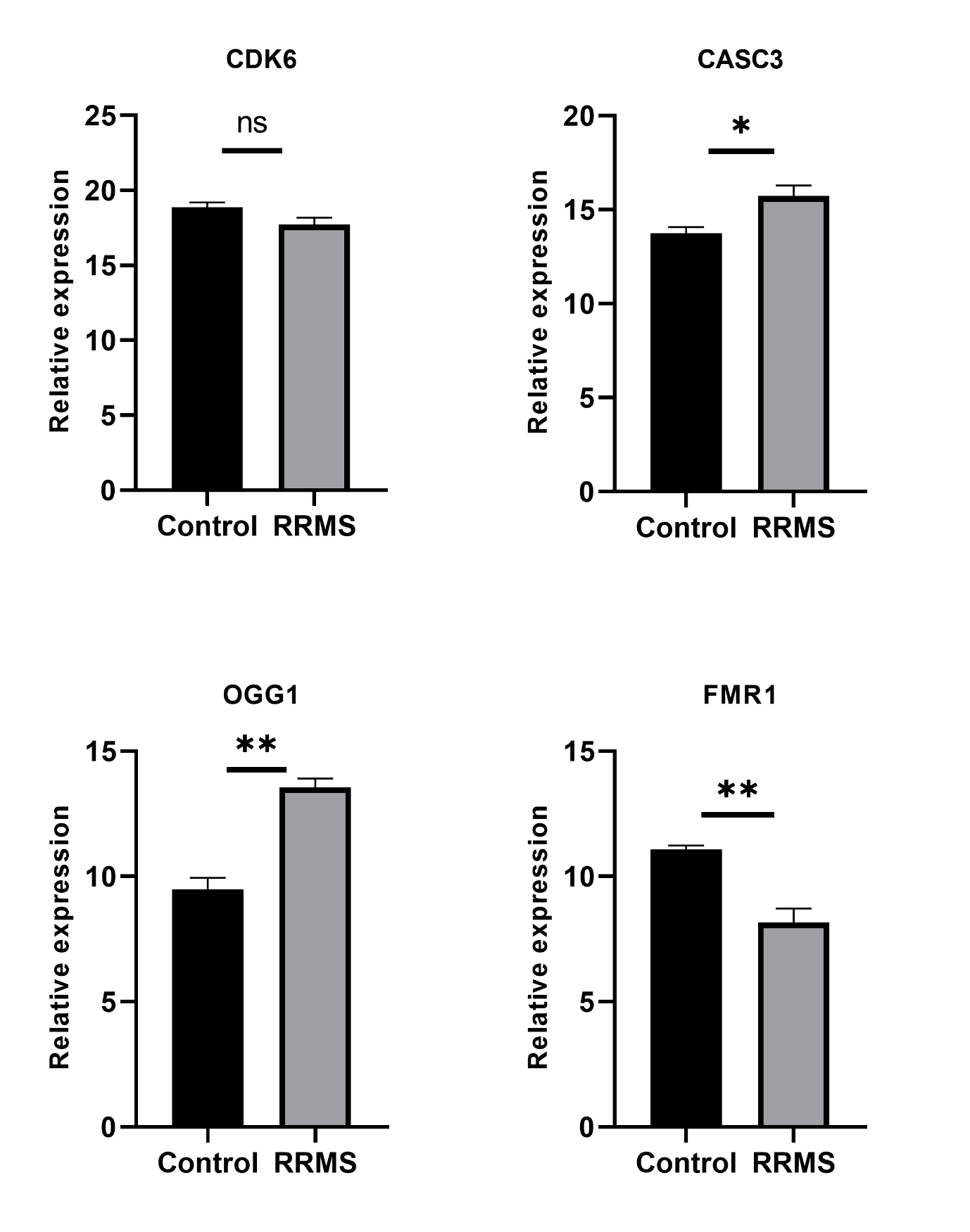


Figure 2.

Subgroup analysis of selected genes in RRMS. Students t test was carried out with age and gender matched controls. Data expressed as means ± standard error of mean. Asterisks indicate statistical difference (*p ≤ 0.05, ** p ≤ 0.01 and ns: no significant).


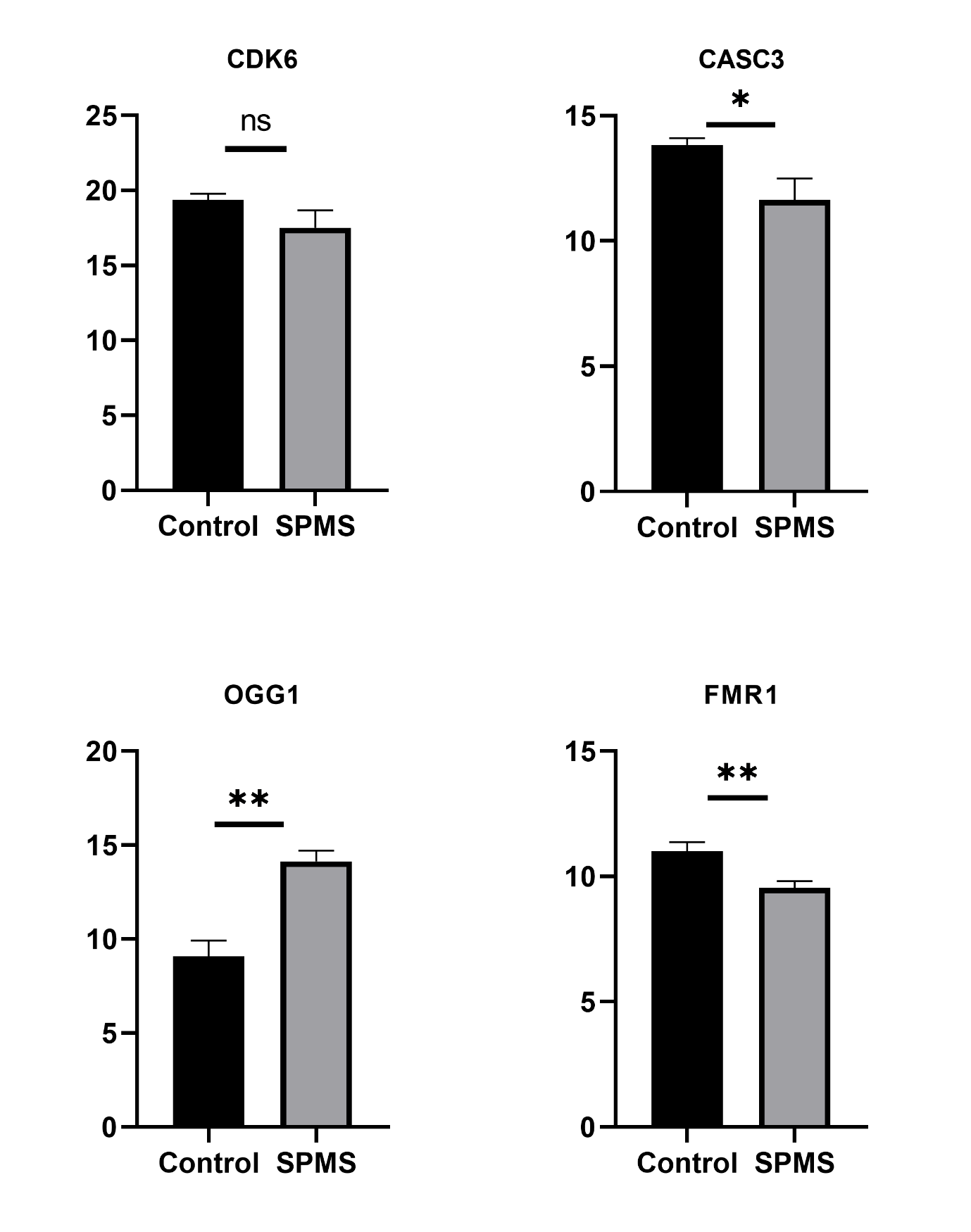


Figure 3.

Subgroup analysis of selected genes in SPMS. Students t test was carried out with age and gender matched controls. Data expressed as means ± standard error of mean. Asterisks indicate statistical difference (*p ≤ 0.05, ** p ≤ 0.01 and ns: no significant).


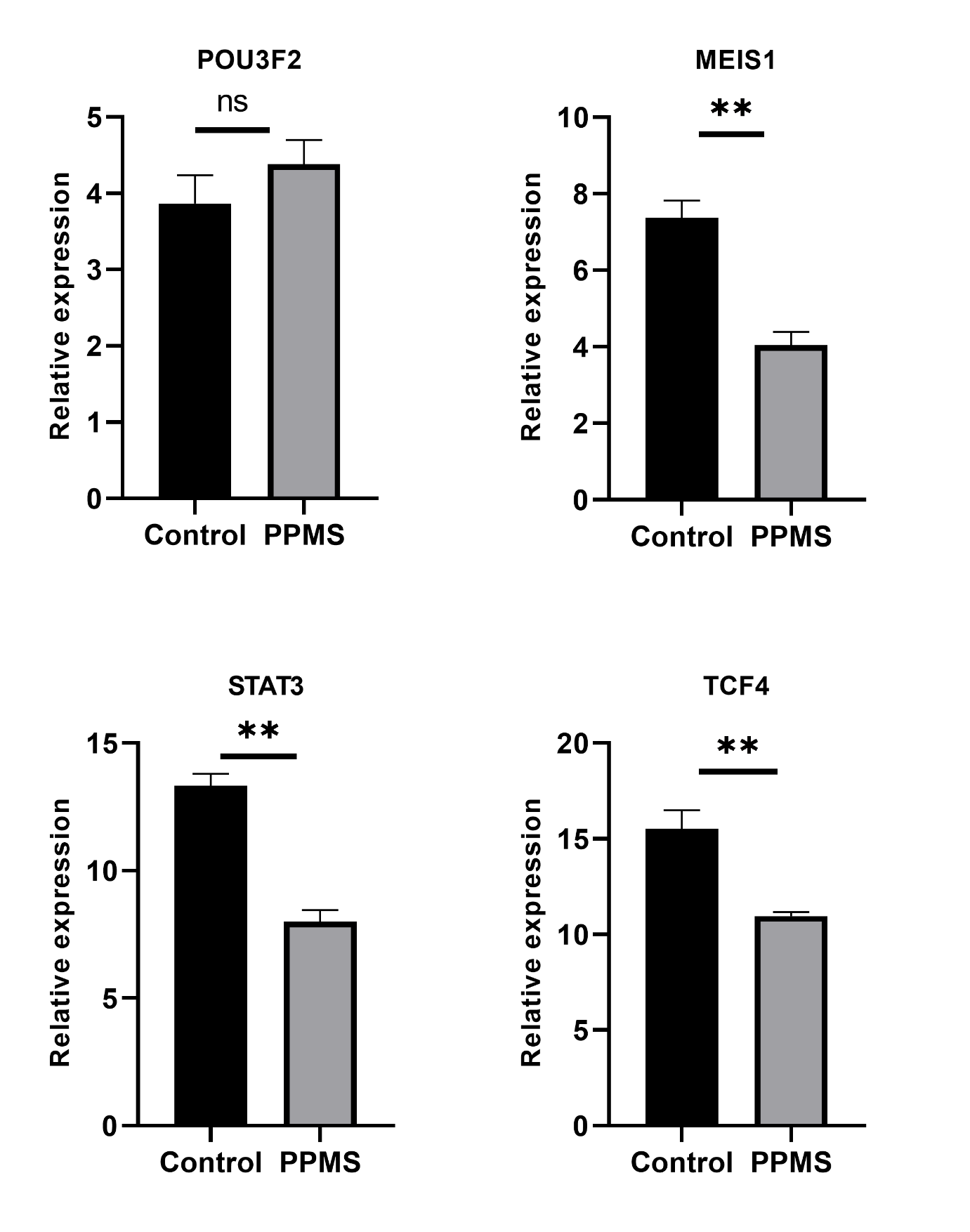


Figure 4.

Subgroup analysis of selected TFs in PPMS. Students t test was carried out with age and gender matched controls. Data expressed as means ± standard error of mean. Asterisks indicate statistical difference (*p ≤ 0.05, ** p ≤ 0.01 and ns: no significant).


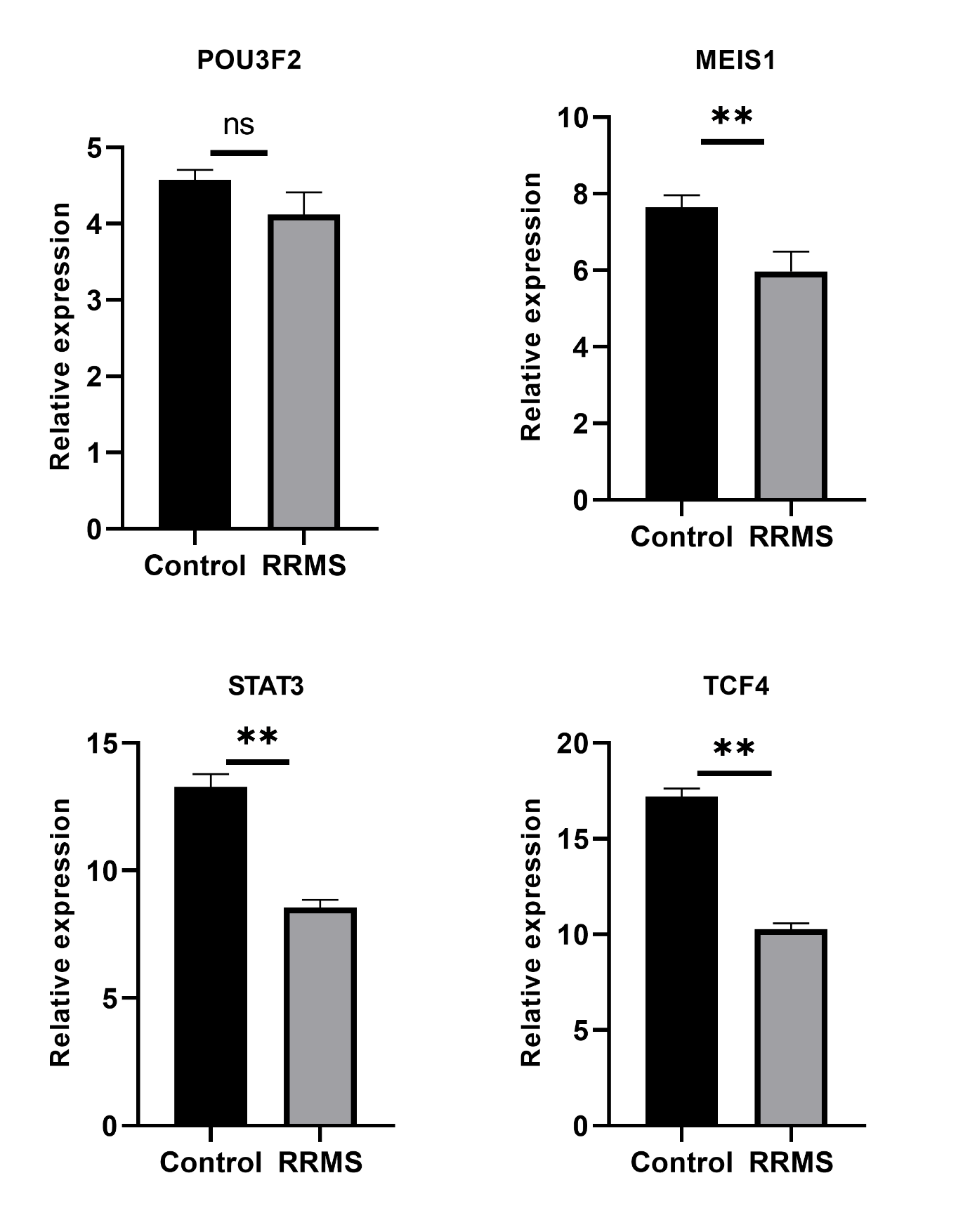


Figure 5.

Subgroup analysis of selected TFs expression in RRMS. Students t test was carried out with age and gender matched controls. Data expressed as means ± standard error of mean. Asterisks indicate statistical difference (*p ≤ 0.05, ** p ≤ 0.01 and ns: no significant).


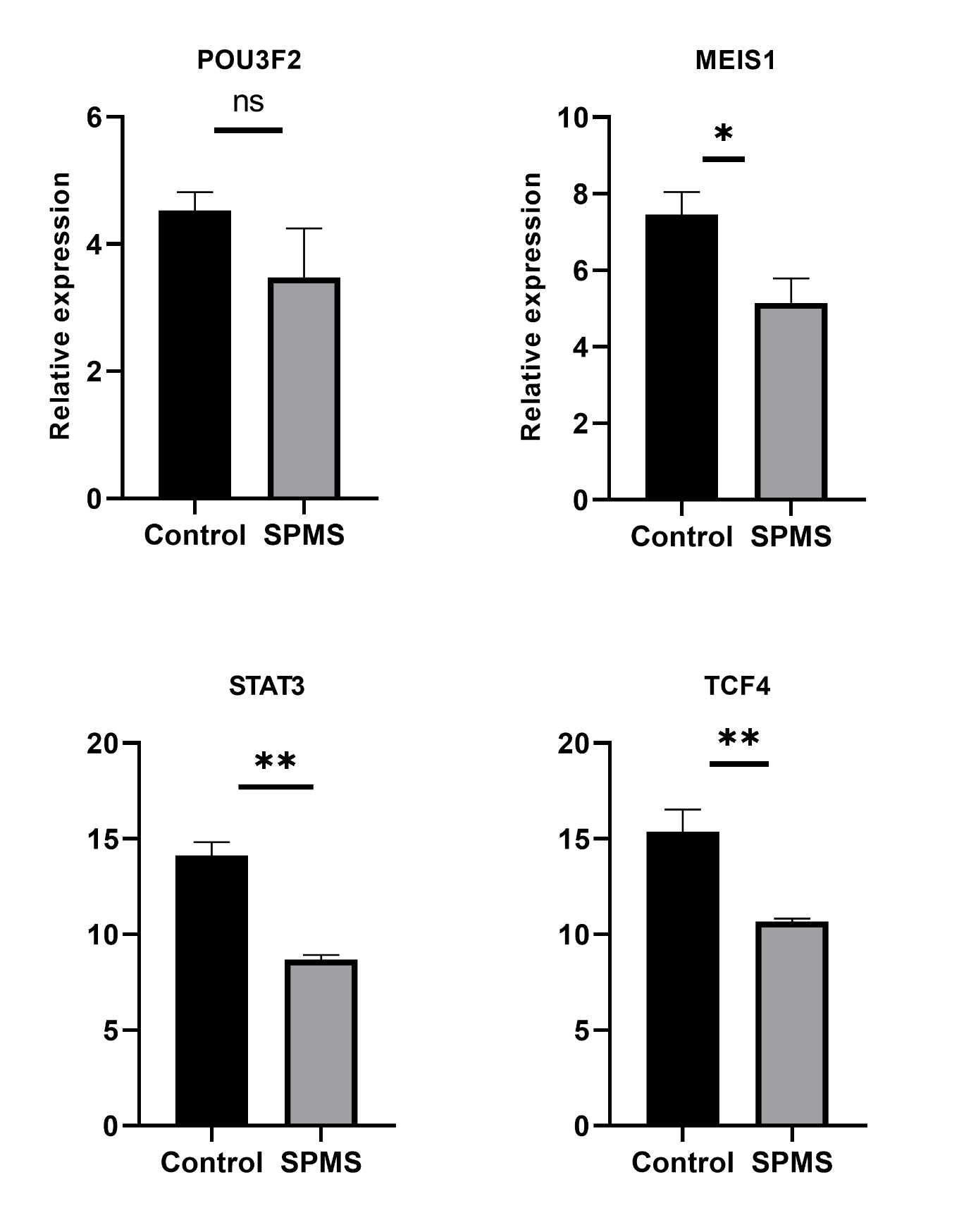


Figure 6.

Subgroup analysis of selected TFs in SPMS. Students t test was carried out with age and gender matched controls. Data expressed as means ± standard error of mean. Asterisks indicate statistical difference (*p ≤ 0.05, ** p ≤ 0.01 and ns: no significant).


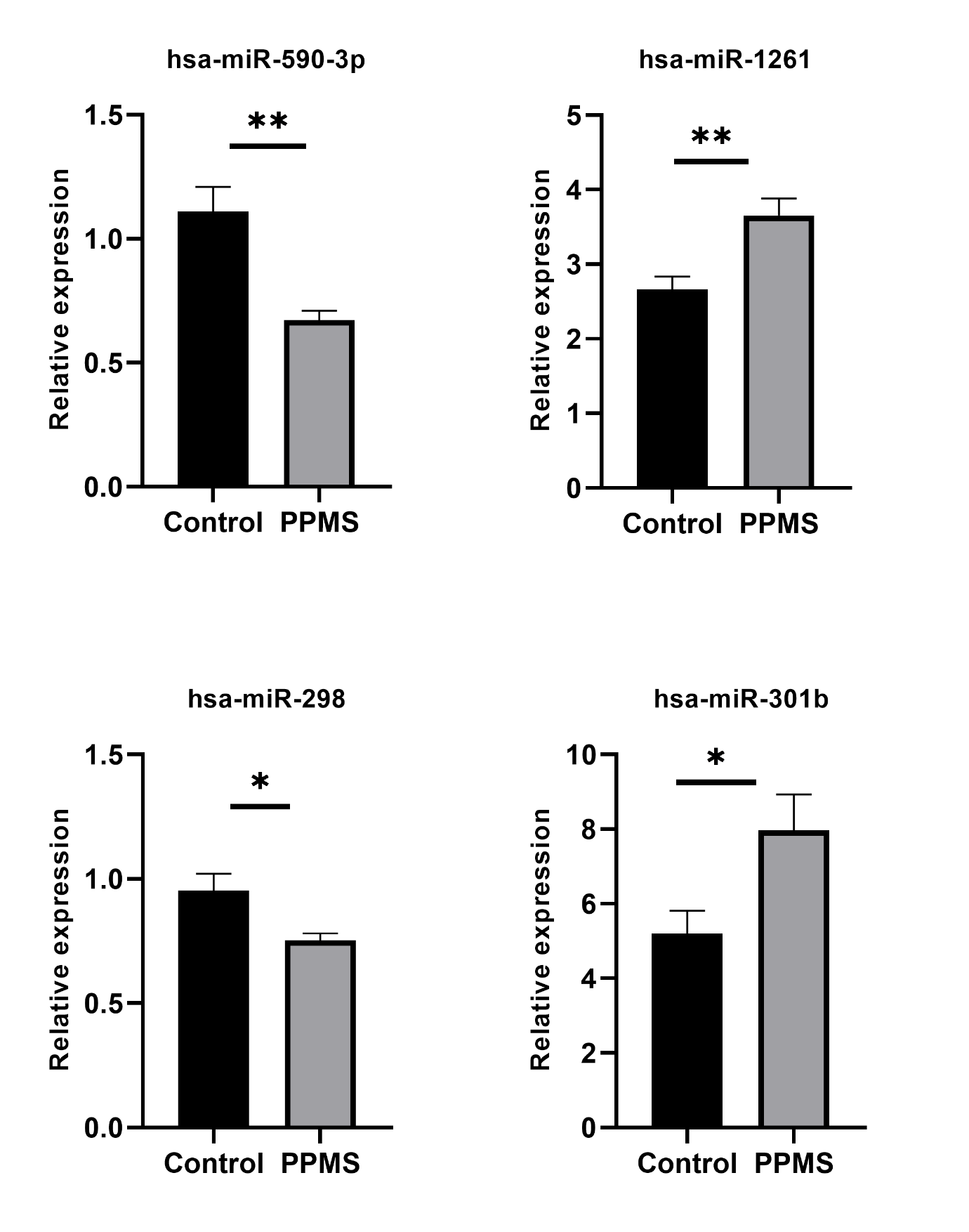


Figure 7.

Subgroup analysis of selected miRNAs in PPMS. Students t test was carried out with age and gender matched controls. Data expressed as means ± standard error of mean. Asterisks indicate statistical difference (*p ≤ 0.05, ** p ≤ 0.01 and ns: no significant).


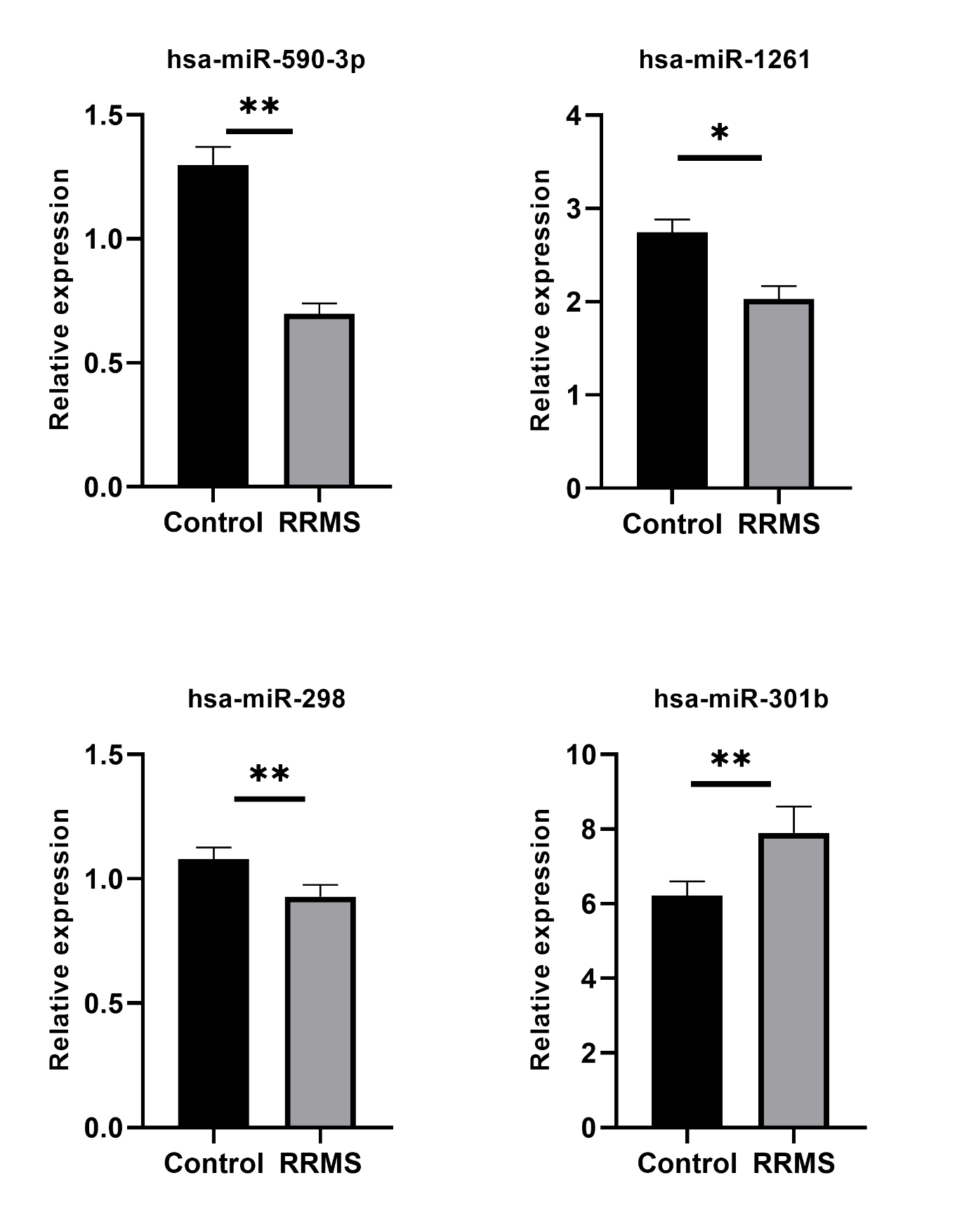


Figure 8.

Subgroup analysis of selected miRNAs in RRMS. Students t test was carried out with age and gender matched controls. Data expressed as means ± standard error of mean. Asterisks indicate statistical difference (*p ≤ 0.05, ** p ≤ 0.01 and ns: no significant).


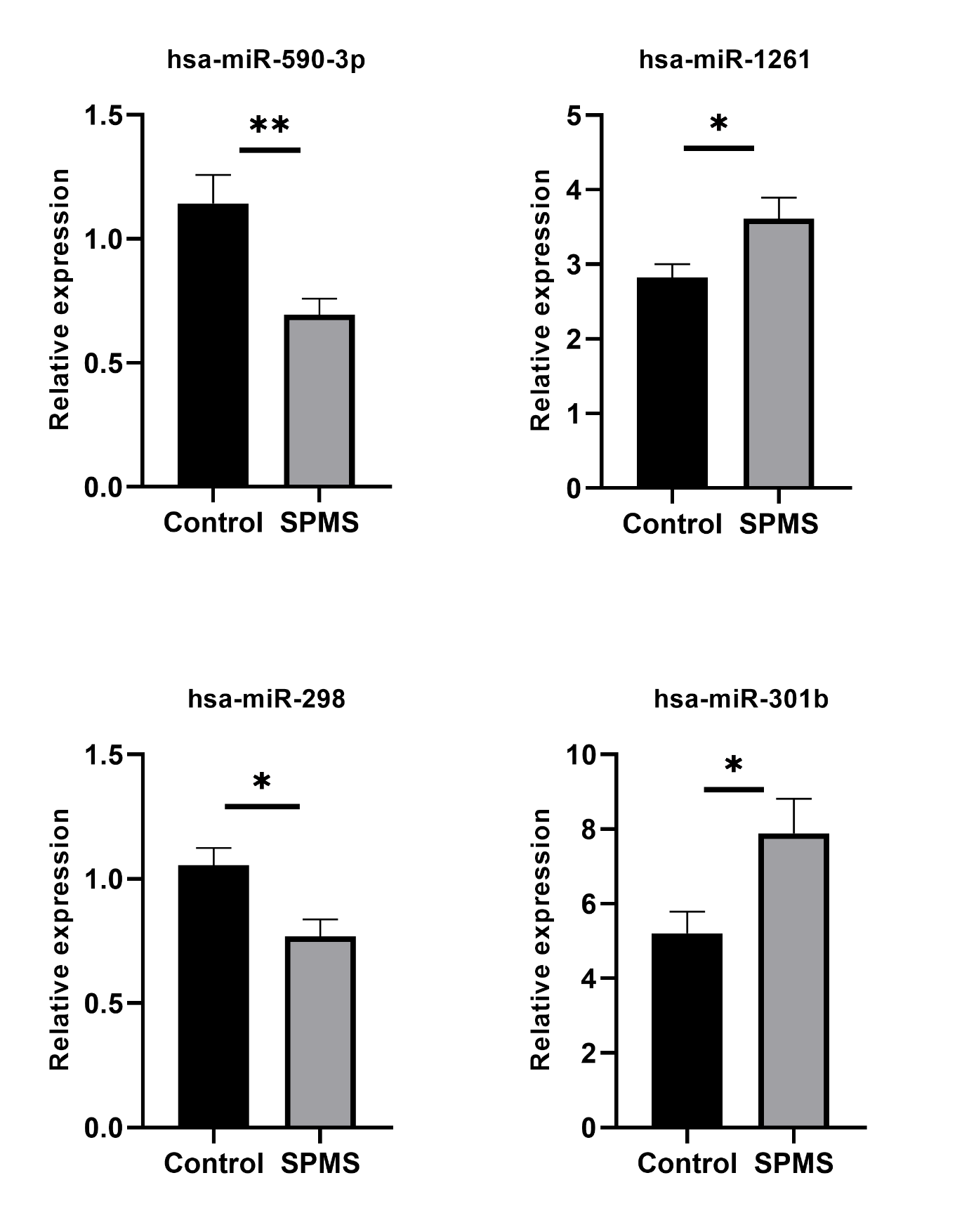


Figure 9.

Subgroup analysis of selected miRNAs in SPMS. Students t test was carried out with age and gender matched controls. Data expressed as means ± standard error of mean. Asterisks indicate statistical difference (*p ≤ 0.05, ** p ≤ 0.01 and ns: no significant).
