# Supplementary material for: Gene Regulatory Networks in Peripheral Mononuclear Cells Reveals Critical Regulatory Modules and Regulators of Multiple Sclerosis
Source: Sci Rep. 2019 Sep 4;9:12732. doi: 10.1038/s41598-019-49124-x (PMC6726613; doi:10.1038/s41598-019-49124-x)
Supplement: Supplementary file 2 — Supplementary information 2 [file 41598_2019_49124_MOESM2_ESM.docx]

**Gene Regulatory Networks in Peripheral Mononuclear Cells Reveals  Critical  Regulatory Modules and Regulators of Multiple Sclerosis**

Perumal Gnanakkumaar, Ram Murugesan, Shiek SSJ Ahmed

Supplement 2: Visual representation of functional enrichment analysis of top-ranked 240 GRNs


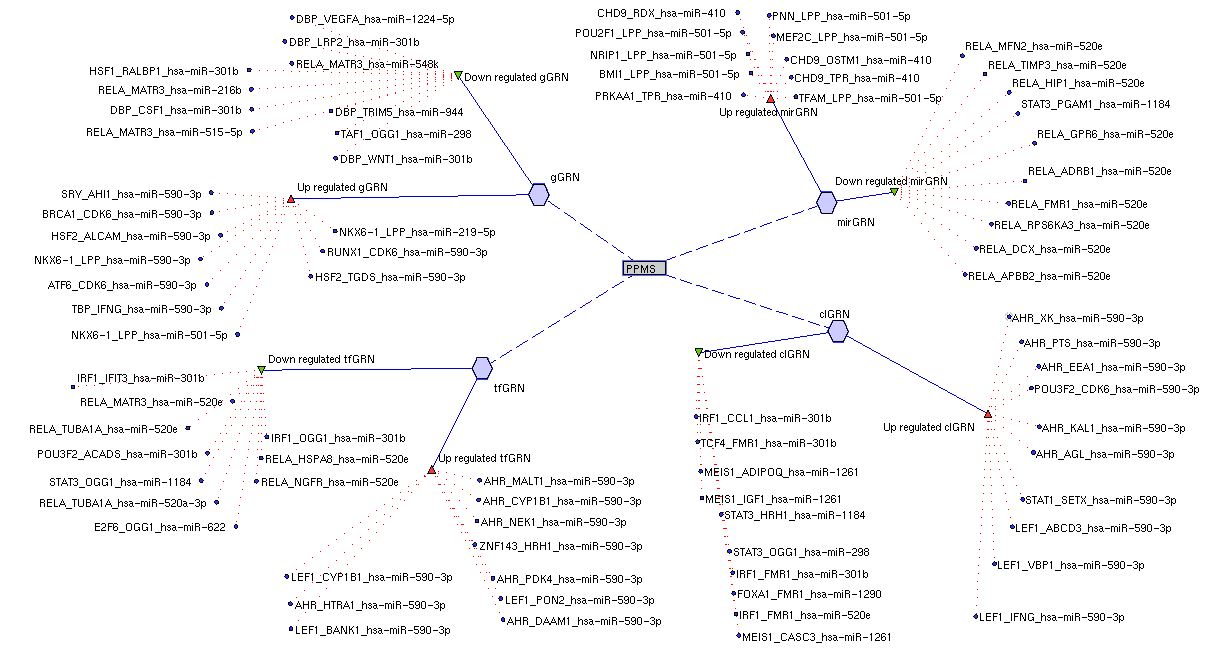


Figure 1. Illustrate differentially regulating top ranked four classes of GRNs in PPMS conditions. Polygon indicates types of GRN and red up faced triangle indicates up regulated GRNs. Similarly, green down faced triangle indicates down regulated GRNs.


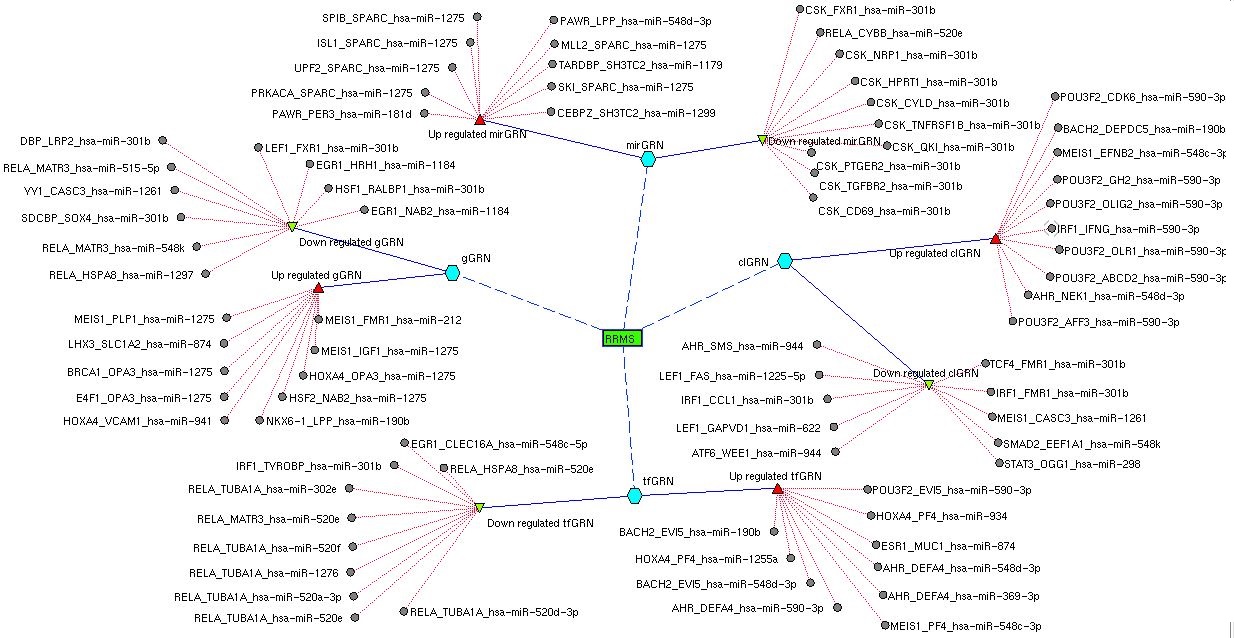


Figure 2. Illustrate differentially regulating top ranked GRNs in RRMS condition. Polygon indicates types of GRN and red up faced triangle indicates up regulated GRNs. Similarly, green down faced triangle indicates down regulated GRNs.


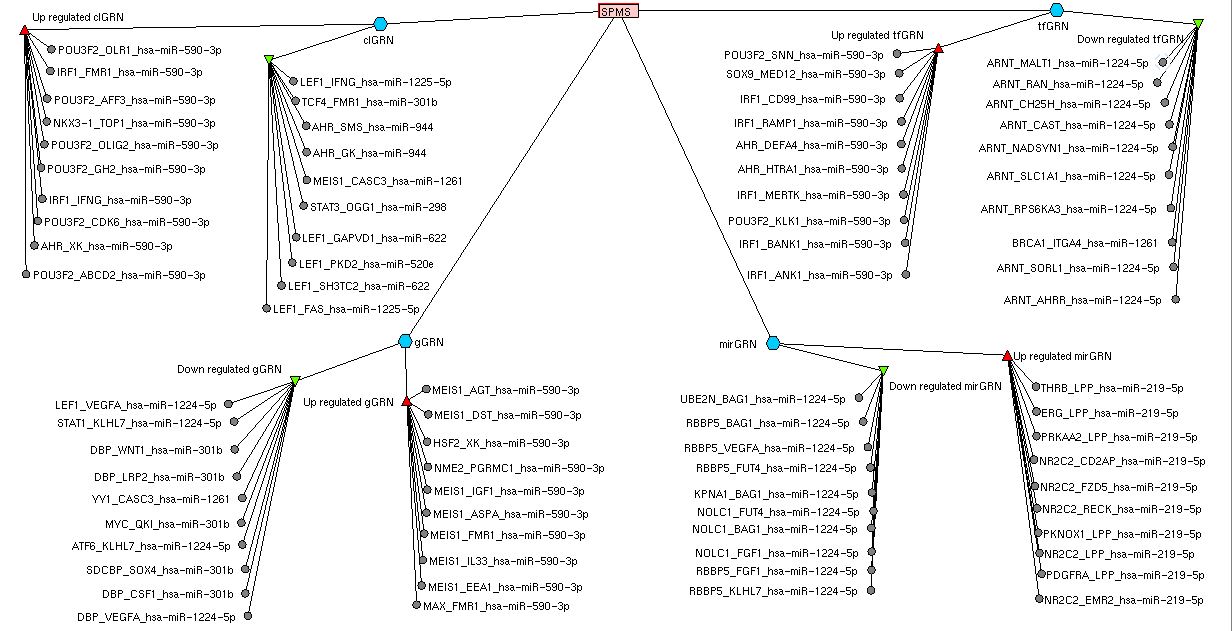


Figure 3. Illustrate differentially regulating top ranked GRNs in RRMS condition. Polygon indicates types of GRN and red up faced triangle indicates up regulated GRNs. Similarly, green down faced triangle indicates down regulated GRNs.


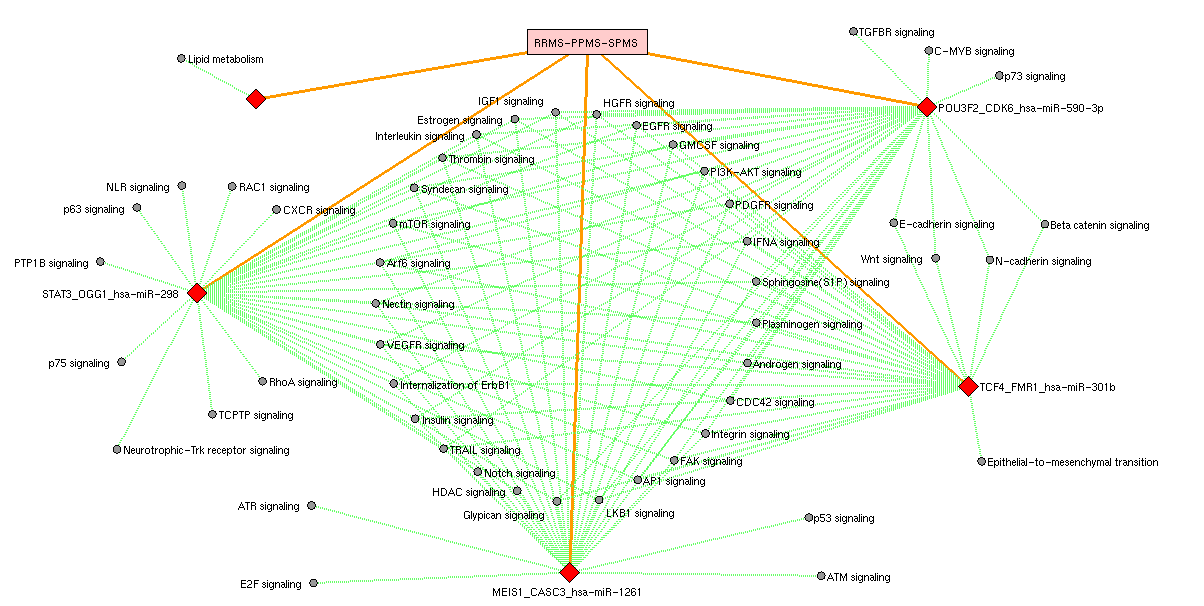


Figure 4 represents pathways regulated five common GRNs between RRMS, PPMS, and SPMS. Red diamond represents GRNs


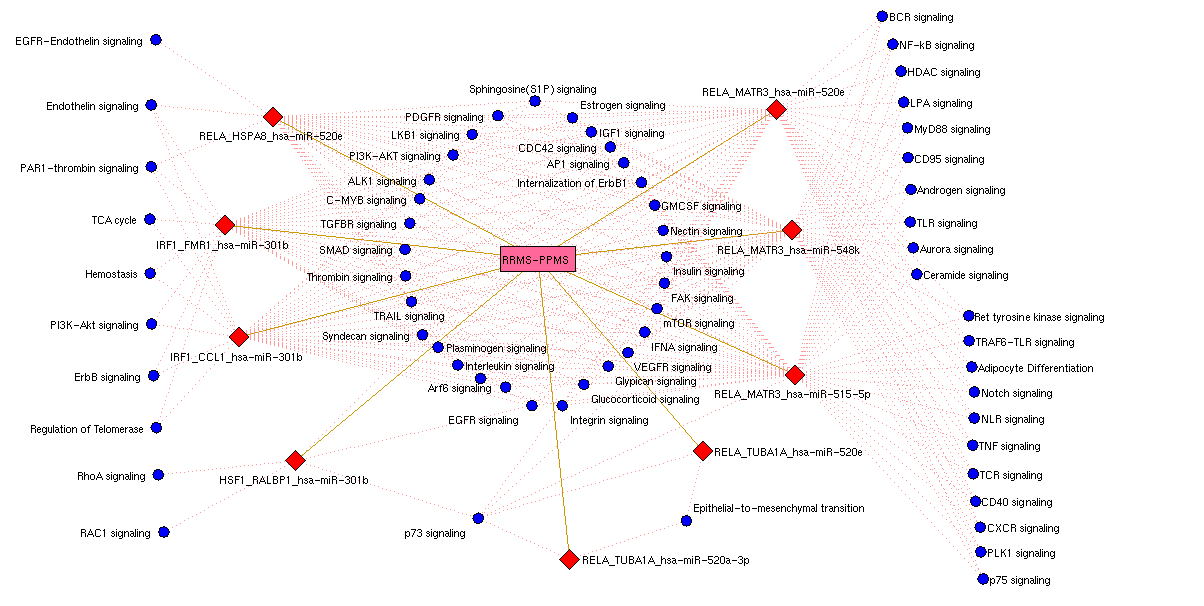


Figure 5 illustrates 63 pathways regulated nine common GRNs between RRMS and PPMS. Red diamond represents GRNs


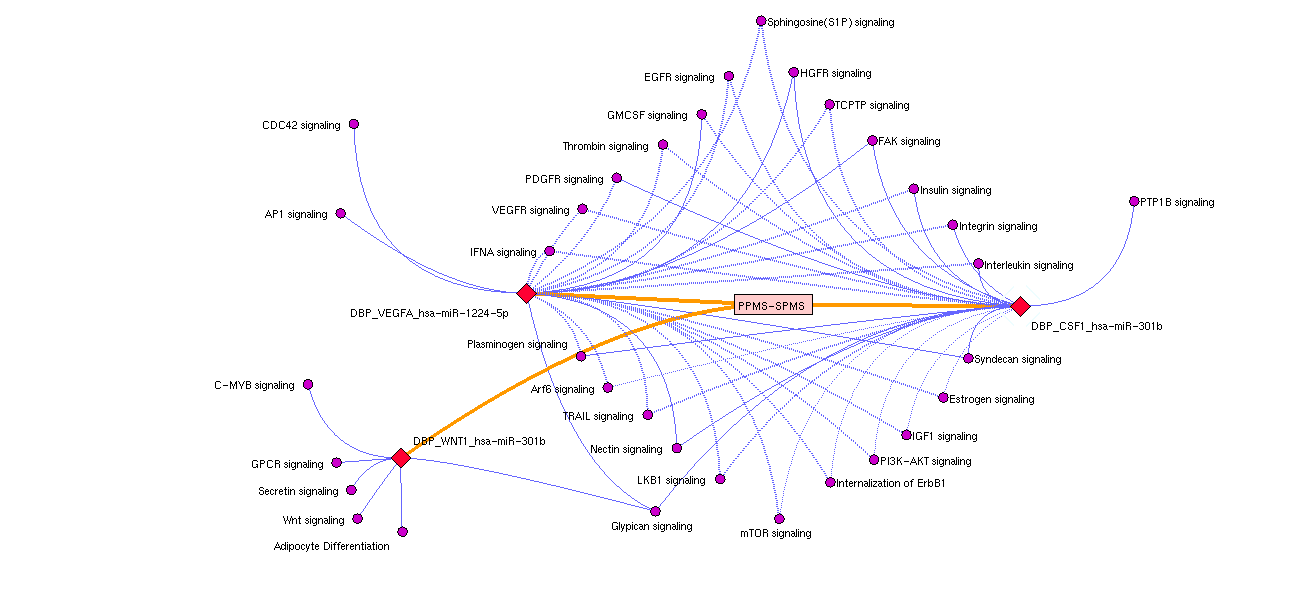


Figure 6 represents 33 pathways regulated three common GRNs between PPMS and SPMS. Red diamond represents GRNs


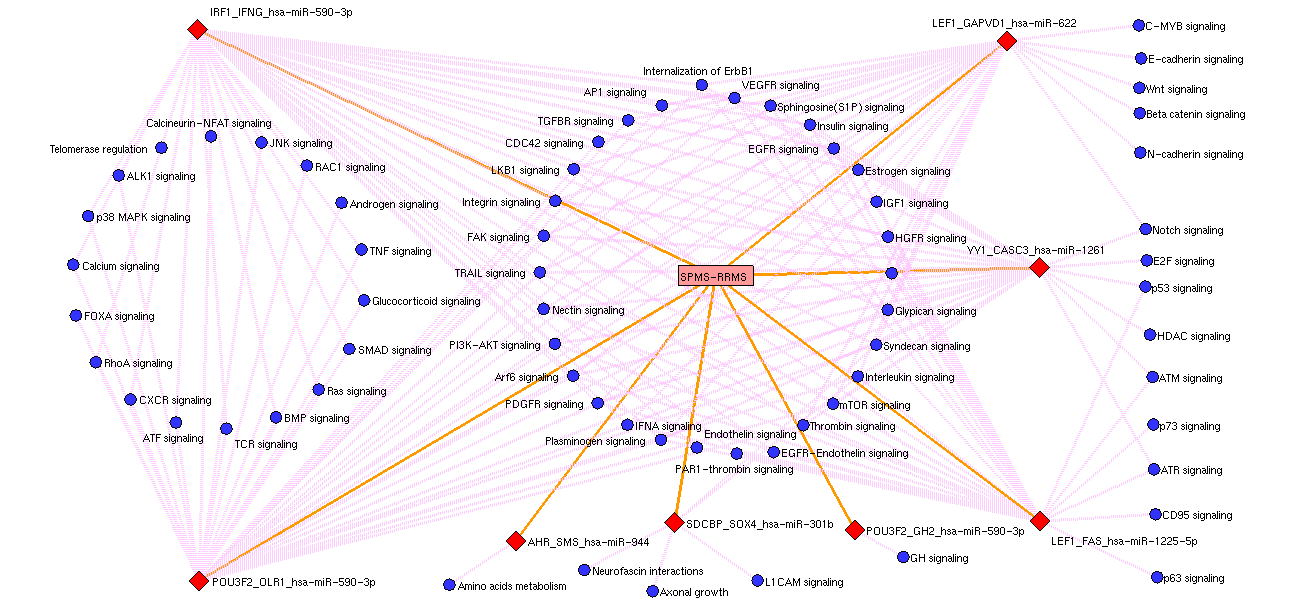


Figure 7 depicts 67 pathways regulated eight common GRNs between SPMS and RRMS. Red diamond represents GRNs
